# Evaluating imputation methods for single-cell RNA-seq data

**DOI:** 10.1186/s12859-023-05417-7

**Published:** 2023-07-28

**Authors:** Yi Cheng, Xiuli Ma, Lang Yuan, Zhaoguo Sun, Pingzhang Wang

**Affiliations:** 1 School of Intelligence Science and Technology, Key Laboratory of Machine Perception (MOE), Peking University, Beijing, 100871 China; 2 Department of Immunology, NHC Key Laboratory of Medical Immunology (Peking University), School of Basic Medical Sciences, Peking University Health Science Center, Beijing, China; 3 Peking University Center for Human Disease Genomics, Beijing, 100191 China

**Keywords:** Single cell, scRNA-seq, Imputation, Clustering

## Abstract

**Background:**

Single-cell RNA sequencing (scRNA-seq) enables the high-throughput profiling of gene expression at the single-cell level. However, overwhelming dropouts within data may obscure meaningful biological signals. Various imputation methods have recently been developed to address this problem. Therefore, it is important to perform a systematic evaluation of different imputation algorithms.

**Results:**

In this study, we evaluated 11 of the most recent imputation methods on 12 real biological datasets from immunological studies and 4 simulated datasets. The performance of these methods was compared, based on numerical recovery, cell clustering and marker gene analysis. Most of the methods brought some benefits on numerical recovery. To some extent, the performance of imputation methods varied among protocols. In the cell clustering analysis, no method performed consistently well across all datasets. Some methods performed poorly on real datasets but excellent on simulated datasets. Surprisingly and importantly, some methods had a negative effect on cell clustering. In marker gene analysis, some methods identified potentially novel cell subsets. However, not all of the marker genes were successfully imputed in gene expression, suggesting that imputation challenges remain.

**Conclusions:**

In summary, different imputation methods showed different effects on different datasets, suggesting that imputation may have dataset specificity. Our study reveals the benefits and limitations of various imputation methods and provides a data-driven guidance for scRNA-seq data analysis.

**Supplementary Information:**

The online version contains supplementary material available at 10.1186/s12859-023-05417-7.

## Background

Advances in single-cell RNA sequencing (scRNA-seq) technologies have enabled the exploration of the transcriptome at the resolution of individual cells [1]. This can potentially reveal heterogeneity and diversity among different cell types [2]. However, despite improvements in experimental protocols, various technical factors lead to substantial noise in scRNA-seq data. In addition, the low transcript capture efficiency and low sequencing efficiency may result in a high frequency of zero or low read counts, defined as dropout events [3]. These can corrupt scRNA-seq data and hinder downstream analyses, such as novel cell type identification and marker gene analysis, which rely heavily on data quality.

Recently, many studies have reported promising advances in the field of single-cell omics, highlighting the importance of single-cell data analysis [4, 5]. In particular, various imputation approaches have been introduced to resolve the problem of dropouts. Some methods assume statistical models underlying the observed expression values, and handle the dropouts with the help of the assumed model [6–8]. Some impute the dropouts through deep learning models [9, 10]. Some combine the deep models with statistical assumption [11, 12]. Besides, some methods are based on network analysis [13, 14], similarity learning [15] or clustering [16].

As the ultimate goal of imputation is to recover true data and gain more reliable biological insights, it is essential to determine whether these methods can aid in subsequent analyses, such as discovering the cell clusters, and determining whether these clusters can be discriminated by marker genes and represent meaningful cell types [17–19]. Moreover, although most methods have exhibited good performance in a range of fundamental analysis tasks, it has been pointed out that imputation may introduce false-positive results [20]. Therefore, there is an urgent need for an unbiased evaluation of imputation methods, and guidance on how to select suitable methods for different data applications.

In this study, we conducted a systematic evaluation for 11 known or adapted imputation methods on 12 real datasets and 4 simulated datasets, based on numerical recovery, cell clustering and marker gene analysis.

We first evaluated these methods from the perspective of numerical recovery, and calculated imputation errors to directly demonstrate their ability to recover true expression levels. We then evaluated the methods on the cell clustering task, to determine their ability to recover and enhance the underlying clusters within the original data. We paid more attention to evaluating the methods based on marker gene expression, because the investigation of marker genes is an excellent way to determine actual biological significance. This study reveals the benefits and limitations of various imputation methods, and provides data-driven guidance for scRNA-seq data analysis.

## Results

### Performance in the numerical recovery of scRNA-seq data

The initial aim of imputation is to impute the dropouts in scRNA-seq data to approximate the true expression values. Therefore, it is a direct way to measure the numerical difference between the true values and imputed values of different imputation methods, to evaluate the bias distribution and imputation accuracy.

**Fig. 1:**
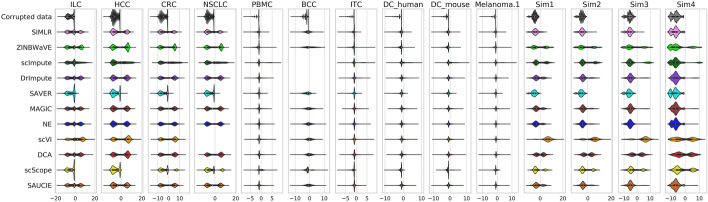
Distribution of log2 normalized differences between the imputed values (or corrupted values) and true values. The performance of different imputation methods for all datasets with raw counts are shown. The differences were calculated by subtracting the true values from the imputed values (or zeros for corrupted data). Positive differences were normalized to $\log _2(value + 1)$ and negative differences were normalized to $-\log _2(-value + 1)$

On real datasets, including ILC, HCC, CRC, NSCLC, PBMC, BCC, ITC, human and mouse DCs and Melanoma.1 (Table 1), most methods tended to slightly underestimate expression values (Fig. 1). Furthermore, on Smart-Seq2 (and Smart-Seq) datasets, some methods, such as SAVER and scScope, significantly underestimated (like the corrupted data) while others, such as DCA and scVI, significantly overestimated expression values. Moreover, some methods, resulted in extremely large expression values, such as scImpute on HCC, CRC, NSCLC, PBMC, DC_mouse and Melanoma.1, and scVI on ILC and HCC. On simulated datasets (Sim1 to Sim4), most methods, especially SAVER and SIMLR, significantly underestimated expression values, while scVI again overestimated expression values, and resulted in extremely large expression values for all simulated datasets.

**Table 1:** Details of all the datasets

Dataset in the study	Source	Description	Data Size$$^{*}$$	Clusters	Sparsity Rates	Original data type$$^{**}$$	Protocol
ILC [27]	GSE70580	Human tonsil Innate lymphoid cells (ILCs)	26087 $\times$ 647	4	87.2%	Raw count	Smart-Seq2
HCC [28]	GSE98638	T cells from hepatocellular carcinoma (HCC)	14127 $\times$ 4050	11	75.0%	Raw count	Smart-Seq2
CRC [29]	GSE108989	T cells from colorectal cancer (CRC)	12547 $\times$ 8496	20	71.6%	Raw count	Smart-Seq2
NSCLC [30]	GSE99254	T cells from non-small cell lung cancer (NSCLC)	12415 $\times$ 9051	16	75.9%	Raw count	Smart-Seq2
PBMC$$^{***}$$	–	Peripheral blood mononuclear cells (PBMCs)	14219 $$\times$$ 5356	5	94.8%	Raw count	Chromium
BCC [31]	GSE123813	Single cells from basal cell carcinoma (BCC)	1000 $\times$ 50026	19	55.9%	Raw count	Chromium
ITC [32]	GSE124731	Human innate T cells (ITCs)	13260 $\times$ 2005	7	93%	Raw count	Chromium
DC_human [33]	GSE137710	Human splenic dendritic cells (DCs)	14064 $\times$ 4406	7	85.6%	Raw count	Chromium
DC_mouse [33]	GSE137710	Mouse splenic dendritic cells (DCs)	12699 $\times$ 4432	7	84.6%	Raw count	Chromium
Melanoma.1 [33]	GSE137710	Single cells from melanoma	15292 $\times$ 8612	7	92.1%	Raw count	Chromium
Melanoma.2 [34]	GSE72056	Single cells from melanoma	22280 $\times$ 4636	7	80.2%	TPM	Smart-Seq2
BRCA [35]	GSE75688	Single cells from breast cancer (BRCA)	27420 $\times$ 515	5	79.0%	TPM	Smart-Seq
Sim1	–	–	600 $\times$ 2000	5	30.7%	Raw count	–
Sim2	–	–	600 $\times$ 2000	5	50.6%	Raw count	–
Sim3	–	–	600 $\times$ 2000	5	70.2%	Raw count	–
Sim4	–	–	600 $\times$ 2000	5	89.6%	Raw count	–

$^{*}$ Number of genes $\times$ number of cells. This is the size of data after quality control

$$^{**}$$ TPM, Transcripts Per Kilobase Million

$$^{***}$$https://support.10xgenomics.com/single-cell-gene-expression/datasets/1.1.0/pbmc6k

More importantly, to evaluate the accuracy of the recovered expression values, we focused on the absolute imputation errors of the different methods, and used their median and mean errors as indicators of accuracy. The median error reflects the general performance of the imputation method, and neglects the effect of outlier values, while the mean error takes the outliers into consideration. Additionally, we also evaluated imputation accuracy based on R2 score (Table 5). The ranking of compared algorithms based on R2 score is similar to that based on mean error.

**Table 2:** Input data type and parameter setting of different imputation methods

Algorithm	Version	Input data type$^*$	Parameter Setting
SIMLR [15]	0.1.3	Raw count, TPM	Default
ZINBWaVE [6]	1.6.0	Raw count	Default
scImpute [7]	0.0.9	Raw count	‘Kcluster’ was set to 5 for simulated datasets, 20 for GSE123813 and 10 for the others.$$^{**}$$
DrImpute [16]	1.0	Raw count, TPM	‘ks’ was set to 5:10
SAVER [8]	1.1.1	Raw count, TPM	Default
MAGIC [13]	1.5.2	Raw count, TPM	Default
NE [14]	–	Raw count, TPM	Default
scVI [11]	0.3.0	Raw count, TPM	‘new_n_genes’ was set to the number of genes of each dataset.
DCA [12]	0.2.2	Raw count	Default
scScope [9]	0.1.5	Raw count, TPM	Default
SAUCIE [10]	–	Raw count, TPM	Default

$^*$For scImpute, ZINBWaVE and DCA, only raw counts are allowed for input

$$^{**}$$To ensure that scImpute obtained the same prior knowledge as other methods, we didn’t provide the accurate number of cell types for it

**Fig. 2:**
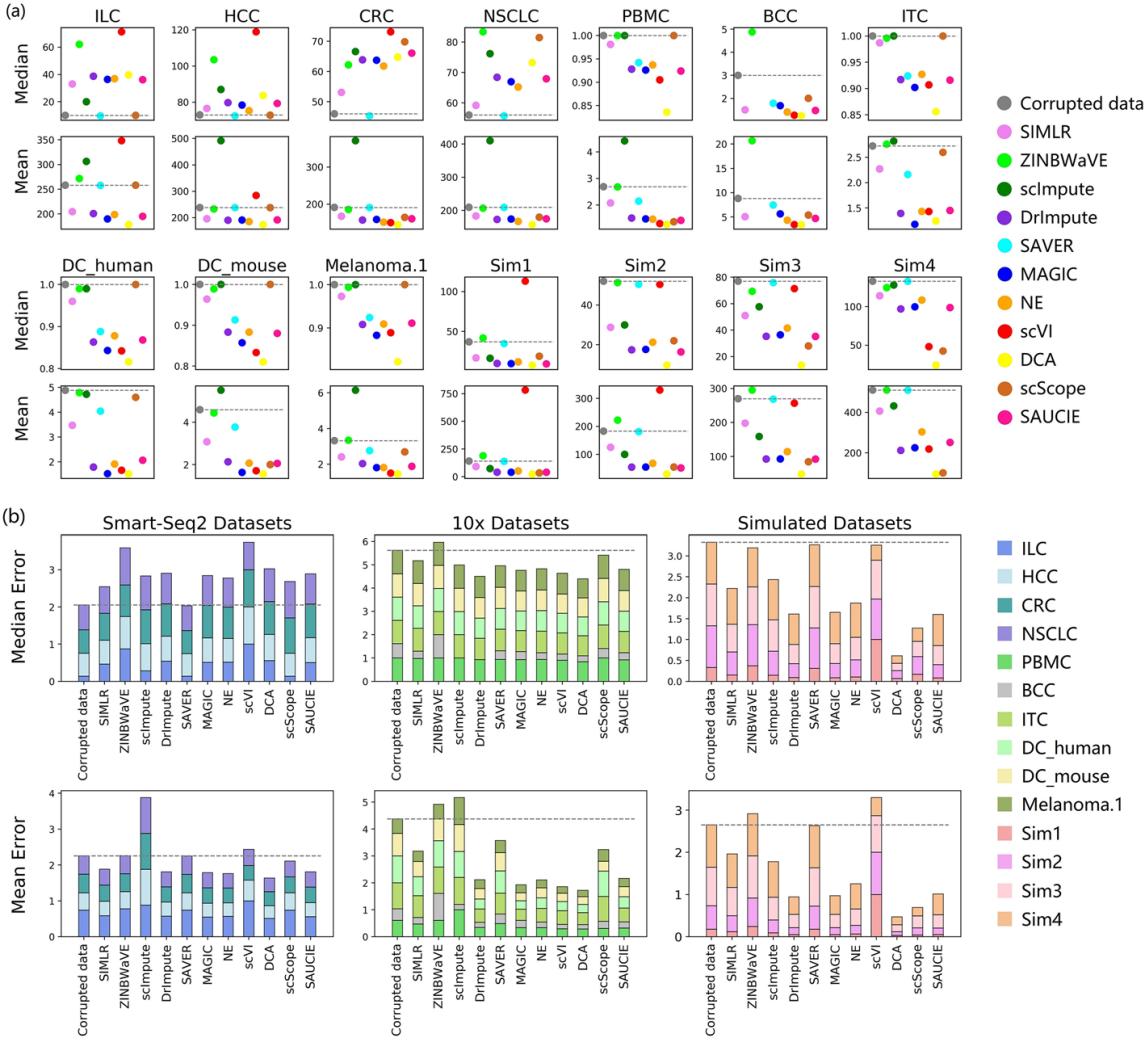
Median and mean imputation errors on raw count datasets. **a** Median imputation error and mean imputation error on each dataset. **b** Normalized total error across the real (Smart-Seq2 and 10x Chromium) and simulated datasets. The normalized error was calculated by dividing the original error by the maximum error on the dataset. Gray dashed baselines indicate the performance before imputation. Lower errors indicate better performance

On real datasets, the effect of the imputation methods varied among the different protocols (Fig. 2). On 10x datasets, most methods explicitly improved the corrupted data. However, on Smart-Seq2 (and Smart-Seq) datasets, imputation can barely accurately recover most of the artificially corrupted values and even introduced more noise (with higher median errors). However, we also found that most methods led to significantly lower mean errors (Fig. 2b), which indicates that large corrupted values were effectively imputed. In general, most methods generally benefited the real datasets, albeit with the addition of some noise. SAVER slightly improved all of the datasets.

On simulated datasets, the imputation methods, especially DCA and scScope, generally performed well (Fig. 2b). Some methods that assume statistical models, such as scVI, ZINBWaVE, and SAVER, led to relatively higher errors than those without statistical assumptions. Given that simulated datasets were generated using Splatter, an scRNA-seq data simulation package that assumes a gamma distribution for the mean expression of each gene and a Poisson distribution based on the read counts in each cell, it may be more difficult for statistical model-based methods to analyze the simulated datasets, which have inconsistent underlying data distributions.

In summary, different imputation methods performed differently in numerical expression recovery. Most methods slightly underestimated expression values on real datasets and significantly on simulated datasets, while SAVER and scScope significantly underestimated on almost all datasets and scVI tended to overestimate expression values. In terms of the recovery accuracy, only SAVER showed a slight, but consistent, improvement on real datasets. On simulated datasets, most methods performed well, especially DCA and scScope, but some statistical model-based methods were less effective.

### Performance in single-cell clustering and visualization

To investigate the effect of imputation on subsequent data analyses, we performed clustering analysis and visualization for data before and after imputation. We implemented single-cell consensus clustering (SC3) and PhenoGraph on both original and imputed data to capture the underlying clustering structure. As the analysis results based on these two methods were relatively consistent, we have only shown the results of SC3.

#### Evaluation of clustering consistency of imputed data

A crucial factor that reflects the effect of imputation on clustering analysis is the consistency between the clusters uncovered from the imputed data and the ground truth. Therefore, the adjusted rand index (ARI) was evaluated for all of the methods.

**Fig. 3:**
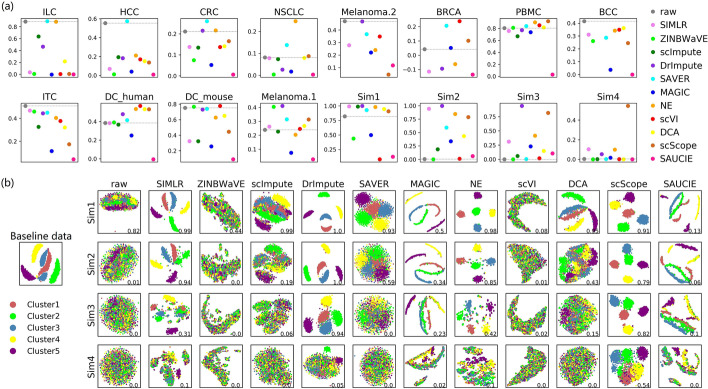
ARI scores based on SC3 clustering. **a** The ARI scores on all of the datasets, with different colors representing various imputation methods, ordered on the *x*-axis in each panel. The dashed baseline corresponds to the ARI score of the clustering results on raw data before imputation. **b** Visualization of the baseline dataset and the simulated datasets derived from it, with different colors representing the simulated clusters. ARI scores are shown in the bottom right corner in each panel. Higher scores indicate better performance

On real datasets, surprisingly, data imputed by most imputation methods had lower ARI scores than those before imputation (Fig. 3a). Most methods did not exhibit expected or satisfactory performance, even on datasets with clear intrinsic clustering structures, such as ILC (Additional file 1: Figure S1), where the ARI score on the raw count data was over 0.8. However, many of the methods did perform well on PBMC, which mainly contains four types of immune cells (T cells, B cells, natural killer (NK) cells, and monocytes) that are easy to distinguish (Additional file 2: Figure S2).

On simulated datasets, most methods performed well on Sim1, which has a dropout rate of approximately 30% (Fig. 3). With the increase in dropout rate from 30% (Sim1) to 90% (Sim4), clustering consistency markedly decreased. However, some methods, such as scScope and DrImpute, still showed better performance. In particular, scScope maintained a distinctively higher ARI score (Fig. 3a) and better clustering visualization (Fig. 3b) than other methods, even though the dropout rate reached approximately 90%. In general, imputation could bring significantly benefits to simulated datasets, although the performance of all methods dropped with increasing sparsity rates.

Thus, it was found that the performance of various imputation methods on real and simulated datasets were quite different. For example, scScope performed very well on simulated datasets, but relatively poorly on many real datasets, such as ILC and Melanoma.2 (Additional file 1, 3: Figures S1 and S3). In general, SAVER, NE, and DrImpute showed better performance on real datasets and, given the biological significance of real datasets, these results should be paid more attention.

We conducted additional experiments to evaluate the clustering results using Purity (Table 3) and NMI (Table 4) metrics. The results showed that the ranking of the compared methods is mostly consistent with that based on ARI metric.

**Table 3:** Purity based on SC3 clustering

Dataset	raw$^{*}$	SIMLR	ZINB-WaVE	scImpute	DrImpute	SAVER	MAGIC	NE	scVI	DCA	scScope	SAUCIE
ILC	0.955	0.510	0.476	0.853	0.760	0.958	0.476	0.957	0.476	0.617	0.478	0.476
HCC	0.720	0.350	0.210	0.404	0.417	0.760	0.270	0.504	0.446	0.415	0.403	0.200
CRC	0.462	0.336	0.272	0.376	0.409	0.517	0.261	0.458	0.388	0.387	0.384	0.174
NSCLC	0.289	0.273	0.149	0.279	0.199	0.353	0.183	0.423	-$$^{**}$$	0.280	0.306	0.152
PBMC	0.928	0.969	0.909	0.955	0.938	0.933	0.859	0.922	0.972	0.920	0.966	0.611
BCC	0.729	0.575	0.583	–	–	0.619	0.294	0.666	0.687	0.662	0.518	0.291
ITC	0.620	0.584	0.563	0.535	0.575	0.555	0.352	0.544	0.539	0.496	0.382	0.265
DC.human	0.787	0.792	0.822	0.815	0.832	0.797	0.726	0.825	0.846	0.846	0.791	0.472
DC.mouse	0.892	0.819	0.904	0.815	0.891	0.900	0.732	0.853	0.854	0.857	0.793	0.607
Melanoma.1	0.896	0.873	0.886	0.855	0.929	0.932	0.779	0.889	0.854	0.890	0.908	0.726
Melanoma.2	0.852	0.766	–	–	0.850	0.787	0.719	0.797	0.873	-	0.535	0.547
BRCA	0.709	0.616	–	–	0.616	0.775	0.652	0.647	0.775	-	0.625	0.616
Sim1	0.924	0.997	0.573	0.996	1.000	0.971	0.701	0.993	0.387	0.978	0.962	0.416
Sim2	0.260	0.978	0.229	0.513	0.999	0.737	0.648	0.939	0.271	0.739	0.894	0.428
Sim3	0.231	0.511	0.228	0.350	0.976	0.244	0.505	0.657	0.282	0.459	0.917	0.369
Sim4	0.242	0.372	0.227	0.239	0.381	0.233	0.275	0.308	0.236	0.240	0.786	0.264

$^{*}$ ’raw’ indicates data before imputation

$$^{**}$$ ’-’ means a method failed to finish imputation

**Table 4:** NMI scores based on SC3 clustering

Dataset	raw$^{*}$	SIMLR	ZINB-WaVE	scImpute	DrImpute	SAVER	MAGIC	NE	scVI	DCA	scScope	SAUCIE
ILC	0.831	0.039	0.014	0.693	0.496	0.841	0.027	0.839	0.013	0.264	0.024	0.019
HCC	0.656	0.203	0.030	0.320	0.286	0.670	0.084	0.382	0.317	0.289	0.255	0.023
CRC	0.380	0.243	0.153	0.294	0.384	0.483	0.150	0.388	0.301	0.346	0.301	0.043
NSCLC	0.162	0.164	0.010	0.172	0.072	0.260	0.050	0.350	-	0.190	0.191	0.021
PBMC	0.821	0.814	0.760	0.780	0.802	0.830	0.703	0.831	0.853	0.833	0.891	0.211
BCC	0.603	0.475	0.460	–	–	0.503	0.113	0.525	0.556	0.546	0.398	0.090
ITC	0.599	0.554	0.585	0.433	0.545	0.556	0.172	0.493	0.466	0.408	0.228	0.087
DC.human	0.557	0.523	0.560	0.542	0.600	0.546	0.379	0.610	0.635	0.615	0.554	0.056
DC.mouse	0.685	0.471	0.698	0.509	0.690	0.690	0.328	0.600	0.666	0.618	0.437	0.171
Melanoma.1	0.557	0.498	0.564	0.466	0.625	0.616	0.208	0.507	0.488	0.536	0.544	0.064
Melanoma.2	0.526	0.422	–	–	0.516	0.412	0.342	0.440	0.517	-	0.054	0.101
BRCA	0.328	0.239	–	–	0.174	0.444	0.302	0.277	0.447	-	0.054	0.189
Sim1	0.780	0.988	0.543	0.982	1.000	0.902	0.594	0.973	0.147	0.925	0.918	0.324
Sim2	0.008	0.928	0.003	0.198	0.996	0.604	0.517	0.849	0.018	0.557	0.878	0.323
Sim3	0.004	0.422	0.002	0.074	0.920	0.010	0.353	0.591	0.032	0.214	0.872	0.166
Sim4	0.007	0.138	0.002	0.007	0.130	0.005	0.034	0.104	0.006	0.004	0.601	0.025

$^{*}$ ’raw’ indicates data before imputation

$$^{**}$$ ’-’ means a method failed to finish imputation

#### Evaluation of cluster coherency of imputed data

The silhouette coefficient is widely used to assess the coherency of clusters, and we therefore used this metric to evaluate the ability of different imputation methods to enhance the clustering structures of data.

**Fig. 4:**
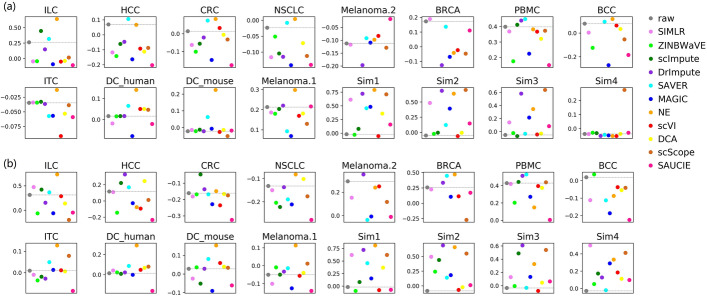
Silhouette scores on all the datasets. **a** Silhouette scores calculated using pre-annotated clusters. **b** Silhouette scores calculated using SC3 clustering results. The dashed baseline corresponds to the silhouette score before imputation. Higher scores indicate better performance

As illustrated in Fig. 4a, most methods slightly recovered the known cluster structures (annotated by the author) of real datasets, but significantly improved those of simulated datasets. This again demonstrated the different performances of methods on real and simulated datasets. On real datasets, only two methods, SAVER and NE, showed relatively good and stable performance, while others did not show satisfactory performance (also shown in Supplementary Figures S4, S5 and S6). In contrast, simulated datasets were improved by most methods.

We also calculated the silhouette coefficient based on the SC3 clustering results, to measure the enhancement of potential cluster structures (Fig. 4b). We found that, on most real datasets, NE, SAVER, DrImpute, and scImpute improved the clustering quality, while others had unstable performance. Besides, on simulated datasets, scScope, DrImpute, NE, and SIMLR clearly enhanced the clusters.

### Evaluation based on marker gene expression and immune cell subsets

As marker genes are not specific to any dataset, they can directly, clearly and unbiasedly characterize cell types from the biological perspective. Therefore, special attention was also paid to evaluation based on marker genes.

**Fig. 5:**
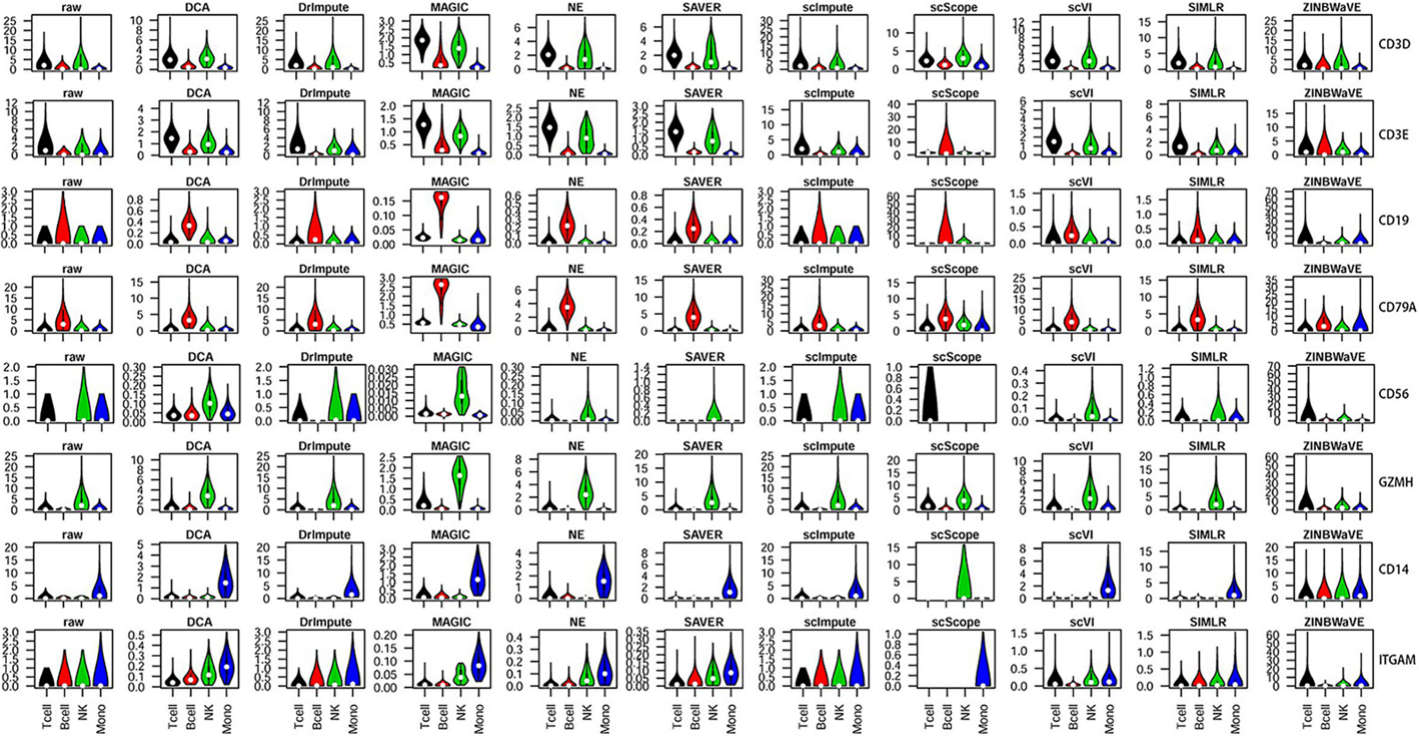
Violin plot illustration of selected marker gene expression in PBMC. Marker genes for various immune cell subsets are shown on the right: CD3D (T-cell surface CD3 delta chain) and CD3E (T-cell surface CD3 epsilon chain) for T cells; CD19 and CD79A (B-cell antigen receptor complex-associated protein alpha chain) for B cells; CD56 and GZMH (granzyme H) for natural killer (NK) cells; and CD14 and ITGAM (integrin subunit alpha M) for monocytes (indicated as ‘Mono’ in the figure)

In general, marker genes showed good discrimination after imputed by DCA, MAGIC, NE, and SAVER on PBMC (Fig. 5). However, some methods were barely able to discriminate different cell subsets based on marker genes. CD3E, which is generally considered to be a T cell marker, although it is also expressed in NK cells at the RNA level, is not expressed in B cells. However, in data imputed by scScope, CD3E showed the strongest expression levels in B cells, whereas it was barely expressed in other cell types. Therefore, imputation may also introduce false-positive results.

**Fig. 6:**
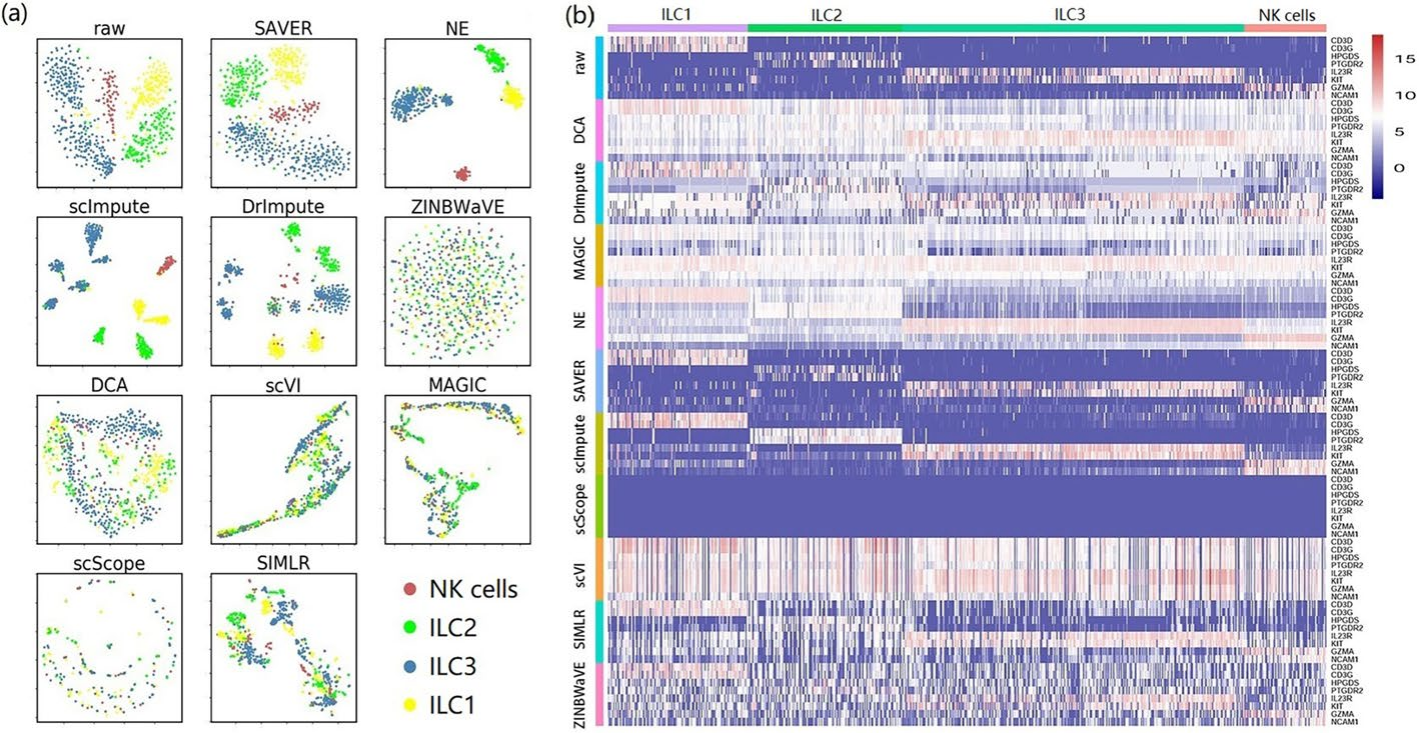
Performance of different imputation methods on ILC. **a** Cluster visualization by t-SNE. Colored cell labels were directly derived from the original study. **b** A heat map illustration of marker gene expression. Expression values were calculated from $$log_2($$raw count $$+1)$$. The color bar indicates expression levels from high (red) to low (blue)

Methods were found to vary greatly in their performance. Some could bring benefits, while others had negative effects instead. ILC comprises three ILC subsets and one NK subset (Fig. 6a). NE explicitly separated the four subsets, and the visualization of SAVER was as clear as the original data. Moreover, scImpute and DrImpute derived novel distinct subtypes for each ILC subset. Based on marker gene expression (Fig. 6b), only SAVER and scImpute performed well, while several other methods performed very unstably. Interestingly, all of the marker genes from data imputed by scScope were shown to be barely expressed (Fig. 6b), which was also observed on PBMC (Fig. 5). After imputed by scVI, ZINBWaVE, and DCA, the patterns of marker gene expression in different subsets were obscured, suggesting that, on ILC, imputation may introduce a large amount of noise.

**Fig. 7:**
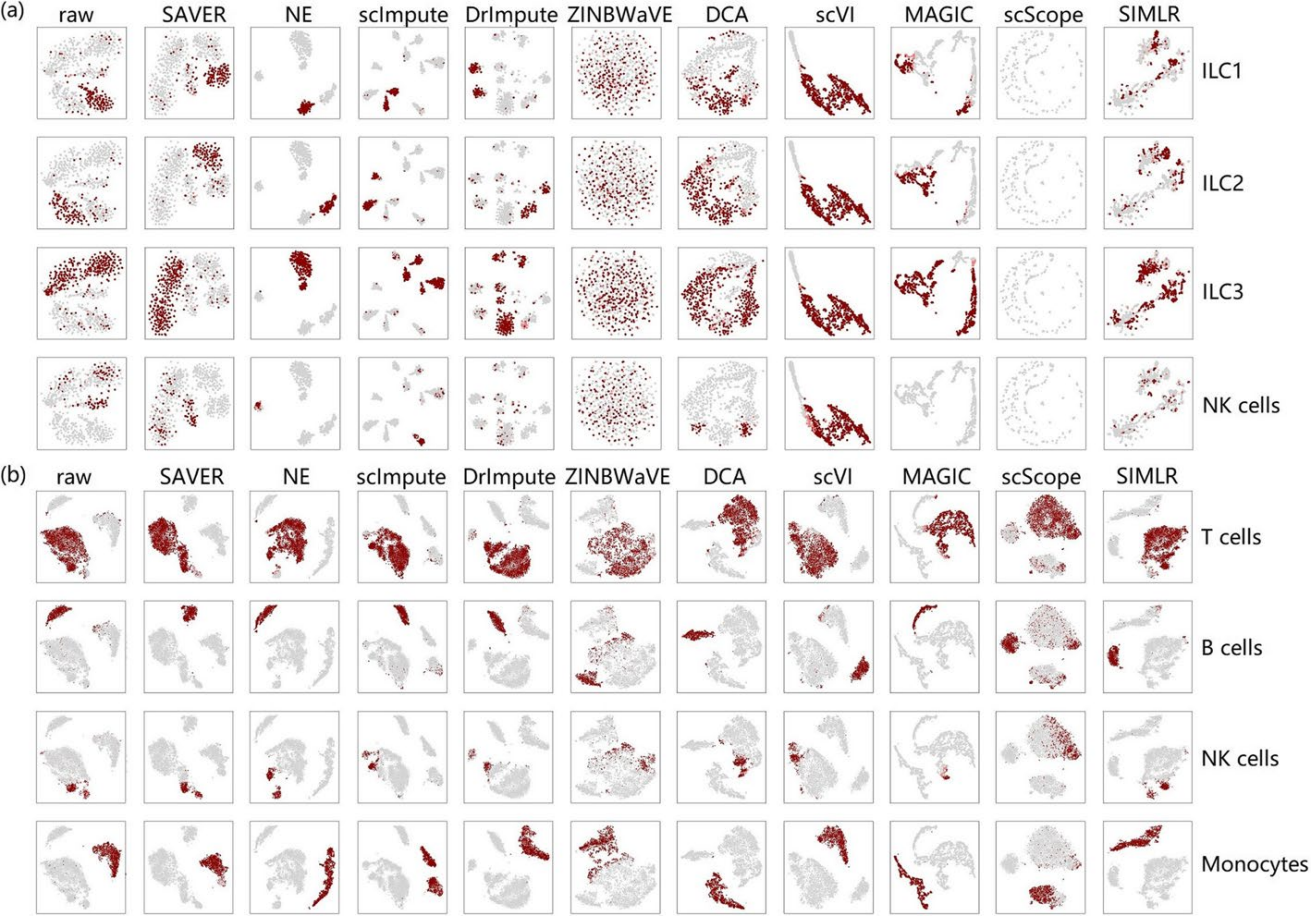
Visualization of marker genes. **a** ILC and **b** PBMC. Cells expressing high levels of marker genes for a specific cell type are highlighted in each panel

Besides, based on the visualization of marker genes (Fig. 7), we found that the performance of different imputation methods was heavily dependent on datasets. For example, on ILC, most methods could not clearly separate different cell types based on marker genes, although some methods, such as SAVER, NE, scImpute, and DrImpute, performed well (Fig. 7a). However, most methods performed very well on PBMC (Fig. 7b), which comprises four major types of immune cells (T cells, B cells, NK cells, and monocytes). Although NK cells slightly overlapped T cells, the original clustering structure was sufficiently distinct. Most recovered data were as clear as the original data, except for data imputed by ZINBWaVE and scScope, where multiple cell types overlapped. Therefore, in terms of marker genes and clustering structure, the performance of imputation methods was dataset-dependent.

**Fig. 8:**
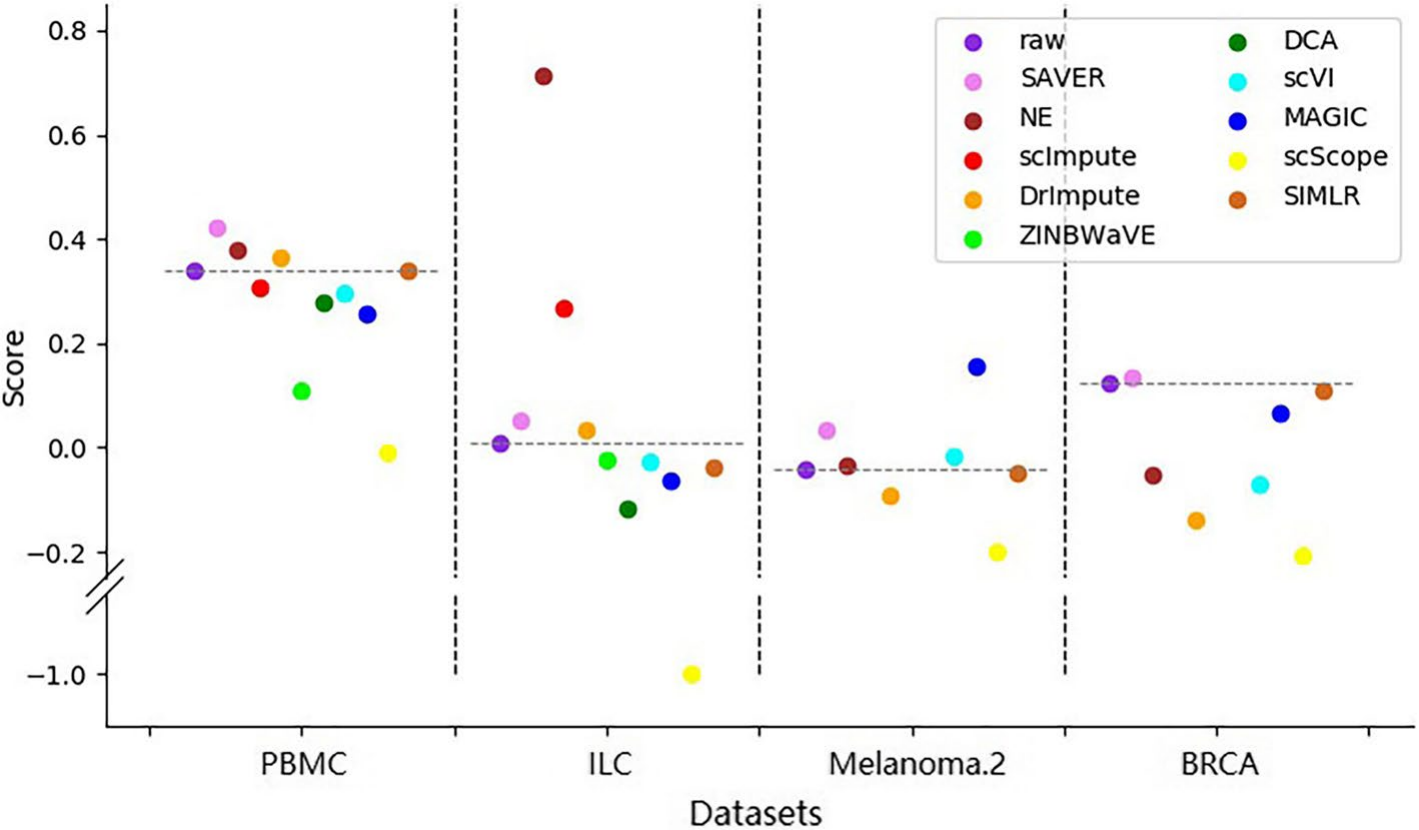
Silhouette scores based on clusters annotated by markers on four datasets. Higher scores indicate better performance

In addition, to evaluate how much the intrinsic structure can be discriminated based on the marker genes after imputation, we also calculated silhouette scores based on the discriminated cell clusters for datasets PBMC, ILC, Melanoma.2 and BRCA (Fig. 8). We found that no matter for datasets that are easy to cluster (e.g. PBMC and BRCA) or that are difficult to separate (e.g. ILC and Melanoma.1), there were always some methods that can improve the original data. SAVER performed the best on these four datasets, outperforming all of the original data, with NE the next best.

In addition to marker gene analysis, one of the most important applications of scRNA-seq data is to identify potential novel cell subsets. Clear clusters mapping to various cell types or subsets are strongly expected, particularly when multiple cell types exist in a dataset.

**Fig. 9:**
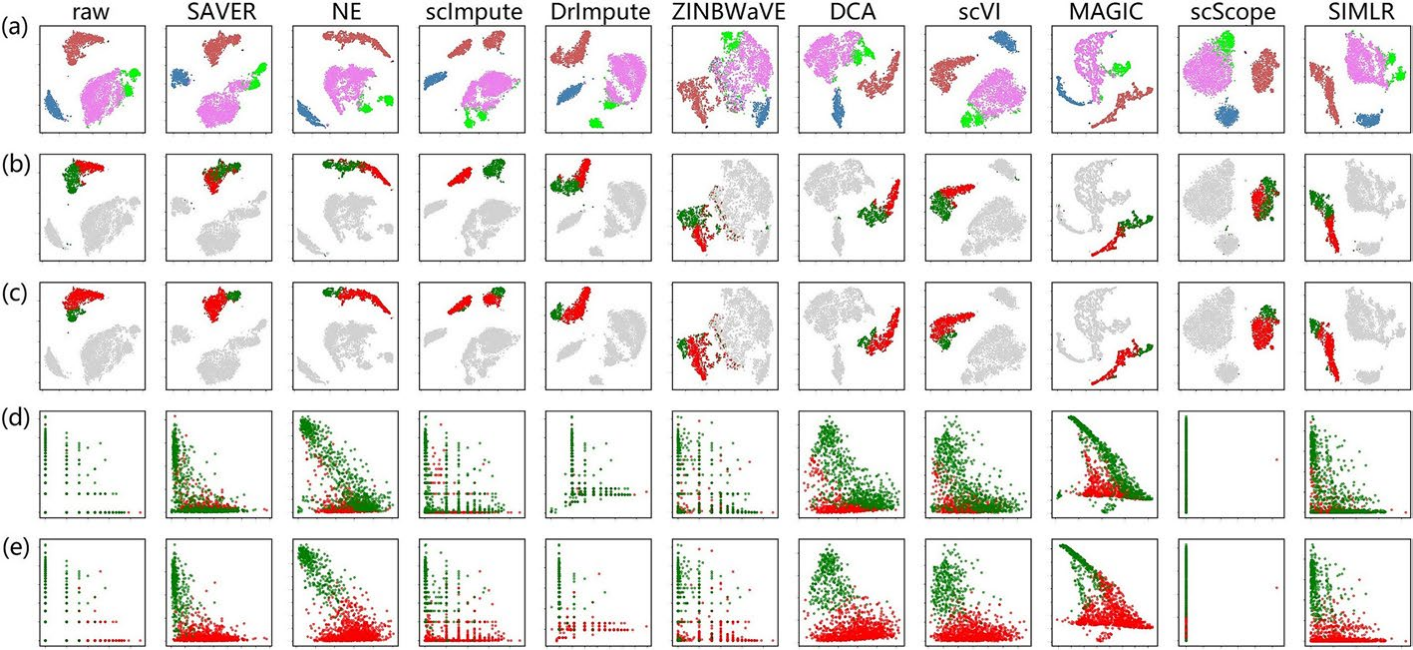
A visual illustration of immune cell subsets based on marker genes. **a** Four main pre-annotated subsets in PBMC. **b** and **c** indicate monocyte subsets, where the labels are derived from the two separated clusters (in red and green) of scImpute and DrImpute, respectively. Scatter plots in (**d**) and (**e**) show a relationship between CD14 (*x*-axis) and CD16 (*y*-axis) expression in the corresponding monocyte clusters, with the same colors from scImpute and DrImpute, respectively

Monocytes are well-recognized in human peripheral blood and are generally categorized into three classes, based on the expression of cell surface markers, denoted CD14 and CD16 (FCGR3A, low affinity immunoglobulin gamma Fc region receptor III-A) [21]. Thus, there are classical (CD14$^{++}$CD16$^{-}$), intermediate (CD14$^{++}$CD16$^{+}$) and non-classical (CD14$^{+}$CD16$^{++}$) monocytes. Interestingly, two separate monocyte clusters were clearly observed after imputed by scImpute (Fig. 9a). When remapped using the subset labels derived from scImpute, monocytes of most other methods, except ZINBWaVE and scScope, showed two separate discernible clusters (Fig. 9b). However, monocyte subsets could not be well discerned from marker gene expression (Fig. 9d), because of the conflicting expression of CD14 and CD16 in different monocyte subsets derived from scImpute.

We further selected the two slightly separated monocyte subsets derived from DrImpute to remap monocytes in data imputed by other methods (Fig. 9c). Based on CD14 and CD16 expression (Fig. 9e), we identified two discriminate monocyte subsets corresponding to the main classical (CD14$^{++}$CD16$^{-}$, in red color) and non-classical (CD14$^{+}$CD16$^{++}$, in green color) monocytes. Intermediate monocytes (CD14$^{++}$CD16$^{+}$) were mainly present in the cluster representing classical monocytes, which makes biological sense. Therefore, DrImpute may perform better in the identification of cell subtypes. This was further supported by ILC, in which ILC3 also showed multiple clusters (Fig. 6a).

Overall, four methods — NE, SAVER, scImpute and DrImpute — improved the original data in the marker gene analysis. scImpute and DrImpute may be beneficial to detect subtle cell types.

## Discussion

Unlike previous benchmarking studies that mainly used cell lines with homogeneous cell populations, this paper evaluates the methods mainly on single cell datasets from real world. Such datasets tend to exhibit greater cellular heterogeneity, introducing higher variability and complexity, making the evaluation more challenging. Additionally, the availability of reference datasets is often more limited compared to well-characterized cell lines, further complicating the evaluation process.

Besides 11 imputation methods mentioned above, we also evaluated some other methods, such as AutoImpute [22]. However, not all methods are suitable for comparison. As AutoImpute first selects and only imputes the 1,000 most variable genes, evaluation tasks, such as numerical recovery, cannot be fairly compared. However, some visualization results about AutoImpute are shown in Additional file 7: Figure S7.

**Fig. 10:**
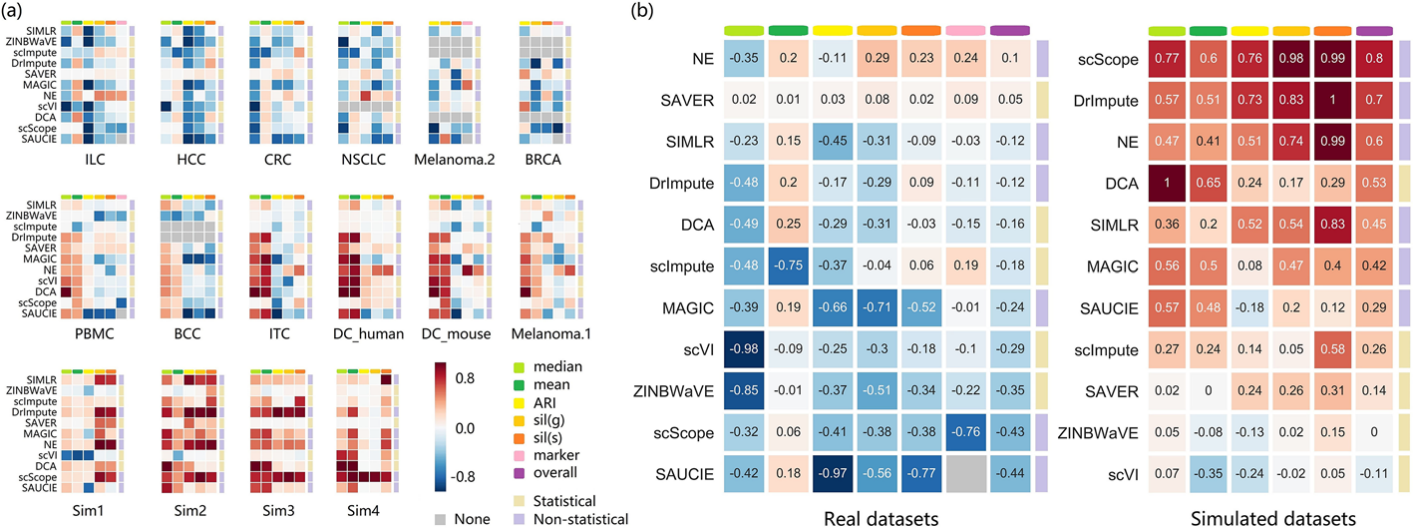
Summary of the performance of the imputation methods. **a** The performance of different methods on each dataset. **b** The overall performance of different methods on real and simulated datasets. In both **a** and **b**, red and blue grids correspond to better and worse performance, respectively. Six metrics on three evaluation tasks are shown in the columns, namely median absolute imputation error ‘median’, mean absolute imputation error ‘mean’, ARI score ‘ARI’, silhouette score based on the ground truth ‘sil(g)’, silhouette score based on SC3 clustering results ‘sil(s)’, and silhouette score based on marker genes ‘marker’. Scores in each column were normalized by subtracting the baseline (data before imputation) score, and then dividing by the difference between the maximum and the minimum score. Opposite scores were used for the ‘median’ and ‘mean’, as lower imputation errors indicate better performance. The methods were categorized as ‘Statistical’ or ‘Non-statistical’, according to their principles. In (**b**), the scores in each grid are the averages across all (real or simulated) datasets. The methods were ranked by the ‘overall’ score, which is a weighted sum of the metrics, with weights of $\frac{1}{6}, \frac{1}{6}, \frac{1}{9}, \frac{1}{9}, \frac{1}{9}, \frac{1}{3}$ for real datasets, and $\frac{1}{4}, \frac{1}{4}, \frac{1}{6}, \frac{1}{6}, \frac{1}{6}$ for simulated datasets, so that each task can make an identical contribution

The evaluation results are summarized in Fig. 10, and reveal that the performance of the methods varied between datasets (Fig. 10a). Moreover, real datasets were only slightly improved by a few methods (Fig. 10b), while simulated datasets were significantly improved by most methods (especially those without statistical models). SAVER and NE are the only methods that improved both real and simulated datasets, while ZINBWaVE and scVI generally brought negative effects. In addition, scScope showed entirely different performances across the two types of datasets.

On the numerical recovery task, almost all of the methods showed a biased estimation of dropout events. Furthermore, statistical model-based methods performed unstably on simulated datasets, which may result from inconsistencies with the assumed models. Additionally, it may be too difficult for imputation methods to achieve accurate numerical recovery. For example, the lowest mean absolute imputation error on ILC exceeded 175 (Fig. 2a), while the mean non-zero expression value of all genes was only approximately 261.

We found that the effects of imputation methods, in terms of median error, varied in different protocols. For most imputation methods, it was difficult to reduce the median error on Smart-Seq2 (and Smart-Seq) datasets, but easy on 10x Chromium datasets (Fig. 2b). This may be due to the different quantification schemes. The former represents read-count only protocols, while the latter is unique molecular identifier (UMI)-based protocol. UMI-based protocols remove duplicates in read counts resulting from polymerase chain reaction cycles during library construction. Thus, to exclude the potential influence of dataset characteristics, another set of datasets, which were recently developed specifically for benchmarking [23], were also evaluated for the top three benchmarking protocols. Similar results (small mean and median errors) were observed on both UMI-based protocols, Quartz-Seq2 and 10x Chromium (Additional file 8: Figure S8a). However, for clustering analysis, the imputation methods did not show apparently different tendencies across different protocols (Figs. 3 and 4), which was further confirmed by the benchmarking datasets (Additional file 8, 9: Figures S8b and S9).

In clustering analysis, based on the ARI metric, only SAVER slightly improved most real datasets, while the other methods generally showed unstable performance. However, on simulated datasets, most methods performed relatively well. Moreover, under the silhouette score based on the ground truth cluster labels, SAVER and NE showed better performance. However, under the silhouette score based on SC3 clustering results, most methods were unstable on real datasets, but performed better on simulated datasets. Overall, no method performed consistently well across all datasets, and some methods even had negative effects on most datasets. Furthermore, it is not easy for imputation methods to improve real datasets, particularly those with biologically homogeneous cell subsets, such as HCC, CRC, and NSCLC. For example, on HCC, in which all of the cells are T cells, most methods performed poorly, as indicated by the ARI and silhouette scores. Thus, how to improve the cluster analysis in highly homogeneous subsets remains a substantial challenge for imputation methods.

Evaluations based on clustering analysis and visualization also suffers from some difficulties, due to the overdependence on the ground truth or the lack of prior knowledge. On one hand, for ARI and silhouette scores based on the known cluster structures, the ground truth was annotated in the original studies. If there is an unknown but significant difference between the ground truth and the true intrinsic clustering structure, evaluation based on the ground truth is of little benefit. Although the ground truth of simulated datasets is accurate, analyses based on simulated datasets are always limited, due to the differences between real and simulated datasets. On the other hand, the silhouette coefficient based on SC3 clustering results, can be used to evaluate imputation methods without ground truth, thus eliminating the errors caused by inexact ground truth. However, evaluation that relies on no prior knowledge would be unreliable. As a result, how to evaluate the effect of imputation on clustering analysis remains to be improved.

During the evaluation, much attention was paid to marker gene analysis, because of its biological significance. Imputation methods are expected to at least recover marker gene expression values. However, our results showed that, different imputation methods had varied performance in marker gene expression and may introduce false-positive signals.

For example, DCA, DrImpute, MAGIC, scVI, and ZINBWaVE introduced a large amount of noise on ILC (Fig. 6b). In addition, on HCC, CRC, and NSCLC, false-positive marker genes were also introduced by these methods, as well as by NE and SAUCIE (Additional file 10: Figure S10). However, more false-positive signals were observed on the Smart-Seq2 datasets (HCC, CRC and NSCLC) than the 10x Chromium dataset (DC_human). Therefore, the benchmarking datasets were further evaluated for marker gene expression.

Five imputation methods were selected according to their performance (Fig. 10), and were evaluated for analysis (Additional file 11, 12, 13: Figures S11, S12 and S13). SAVER showed the best performance across different protocols. scImpute also performed well. DrImpute and NE performed better on UMI-count datasets than the Smart-Seq2 dataset. Therefore, the induction of false-positive marker signals may be involved in protocols, imputation methods and datasets.

Besides, based on marker gene analysis, it appeared that imputation may assist the discovery of potentially novel cell subsets. DrImpute and scImpute have been found to have advantages to identify more sub-clusters (Fig. 9), which may facilitate the discovery of novel subsets. However, they should be used with caution, as the derived subsets have yet to be further validated. It is important to consider whether the clusters are induced by varying sequencing quality or other factors such as batch effect. If these factors have been accounted for, it is ideal to further identify truly reliable marker genes for these new subpopulations. In terms of marker genes, when a cell subtype is divided to subtler sub-divisions, specific marker genes in these sub-divisions will become more difficult to identify. Therefore, datasets with highly homogeneous structures, or those with many subtle sub-populations, would be difficult to impute for most methods, suggesting that imputation challenges remain.

We also evaluated the impact of imputation on gene-gene correlation. Based on some significantly correlated gene pairs from bulk RNA-seq datasets [24], we compared their correlation before and after imputation. We found that some imputation algorithms, such as MAGIC, scVI and scScope, were indeed able to improve the correlation. However, we also discovered that these methods introduced a significant number of false positive signals, which accords with the previous observation [20].

There are some potential improvements for imputation methods. For example, to promote biological discoveries, imputation methods should focus more on the improvement of data in downstream analyses, which is closely related to the method design. Therefore, imputation methods could incorporate the characteristics of scRNA-seq data, such as interactions among genes and connections between cells, to improve their effectiveness. Moreover, with the development of high-throughput sequencing technology, the size of scRNA-seq data will grow rapidly. However, some imputation methods cost a lot of time on some datasets (Additional file 14: Figure S14). Therefore, the scalability and efficiency of imputation methods should be improved, to adapt to future developments and trends. Finally, with the development of single-cell multi-omics methods [25, 26], integrating data from multiple levels will improve imputation performance and the downstream applications.

There are some guidelines for using imputation methods. It is suggestive to try and compare several well-performing imputation methods (such as SAVER and NE), then choose the best-performing one for subsequent tasks. Furthermore, it is essential to understand the purpose of the analysis. For instance, when analyzing the gene-gene correlation, high correlation after imputed by some methods, like MAGIC, should be treated with caution, unless it can be supported by other techniques such as bulk RNA-seq. Additionally, since imputation is dataset-specific, it is crucial to analyze the expression patterns of known marker genes after imputation to determine their validity within the dataset.

## Conclusions

In this study, we conducted a systematic evaluation of 11 imputation methods for scRNA-seq data. The results revealed that the performance of different methods varied across different datasets, suggesting that imputation may have dataset specificity. In particular, based on the experiments evaluating downstream analysis, real datasets were barely improved by most imputation methods. In contrast, simulated datasets were always improved. Furthermore, methods without statistical models had more advantages on simulated datasets.

In general, based on their performance in cell clustering and marker gene analysis, two imputation methods, SAVER and NE, are recommended for downstream analyses. In additional, we recommend DrImpute and scImpute for discovering novel subtle cell types, due to their potential in identifying sub-clusters of single cells.

## Methods

### Datasets and preprocessing

All of the tested datasets [27–35] are shown in Table 1. They vary in size from a few hundred to tens of thousands, with varying sparsity rates (proportion of zero entries) and different numbers of inherent cell subpopulations, thus allowing a comprehensive evaluation of the imputation methods. In addition, all of the real datasets comprise certain types of immune cell subsets, such as T cells, B cells, natural killer (NK) cells, monocytes, dendritic cells (DCs) and innate lymphoid cells (ILCs). For example, dataset PBMC is mainly composed 4 distinct cell types (T cells, B cells, NK cells, and monocytes), while dataset CRC contains 20 highly homogenous cell subsets (12 CD4$^{+}$ T cell subsets and 8 CD8$^{+}$ T cell subsets), which poses different challenges for imputation.

To further evaluate the effectiveness and robustness of the different methods, four simulated datasets with varying proportions of dropouts were synthesized using Splatter [36]. Briefly, a baseline dataset without dropouts was first generated using the default parameters in Splatter. This dataset contains 2000 cells, 600 genes, and 5 clusters. Four datasets with different sparsity rates, ranging from 30 to 90%, were then derived from this baseline dataset.

Quality control of the real datasets was performed before imputation. First, bulk RNA samples within the datasets were removed. Low-quality single cells were then filtered out if the number of expressed genes or the library size exceeded the upper threshold or fell below the lower threshold. The upper threshold was defined as the 75th percentile of all cells plus three times the interquartile range (IQR), while the lower threshold was defined as the 25th percentile minus three times the IQR. Genes that were expressed in no more than two cells were removed.

In dataset BCC, which contains more than 50,000 cells, only the top 1000 genes with the highest expressional variance were retained for imputation, to speed up the calculation. DrImpute and scImpute were not applied to this dataset, as the number of cells exceeds the limit of DrImpute, and the run time of scImpute exceeds our time limit (5 days).

### Numerical recovery of gene expression values

To quantify the numerical recovery of the scRNA-seq data, we measured the imputation error for each imputation method on datasets with raw count data.

Specifically, for simulated datasets, the baseline dataset, which has true expression values that are missing in the four simulated datasets, was treated as the ground truth. Following a similar strategy in scScope [9], two lists, *l* and $l'$, were constructed, in which elements respectively corresponded to the ground truth and the imputed values for all of the dropout entries. The mean imputation error was defined as the mean distance between *l* and $l'$, and the median imputation error was defined as the median distance between *l* and $l'$.

For real datasets, we followed the downsampling strategy used in scVI [11]. We simulated the dropout process by corrupting the real datasets, randomly selecting 10% of the non-zero entries and setting them to zero. We then imputed the corrupted datasets and compared the recovered data with the original data. The imputation error was calculated using the same method as that used for the simulated datasets. We repeated the dropout process ten times for some datasets, and found that the randomness of the dropout process had little effect on the performance of most imputation methods.

### Clustering analysis and visualization

Two clustering algorithms, PhenoGraph [37] and SC3 [38], were used for clustering analysis. Default parameters in SC3 were used, except that ‘gene_filter’ was set to ‘FALSE’ and ‘ks’ was set to the real number of clusters. All of the default parameters in PhenoGraph were used. In addition, to visualize the intrinsic structure of the high-dimensional data, the non-linear dimension reduction method, t-distributed stochastic neighbor embedding (t-SNE) [39], was used, with the parameter ‘perplexity’ set to 50. Before clustering and visualization, all expression values were scaled to $log_2(value+1)$, except when using SUACIE, as it would result in some negative values. All samples were then reduced to 50 dimensions using principal component analysis (PCA).

To compare the consistency between the clustering results and the ground truth or the original label in the corresponding study, we used adjusted rand index (ARI), which is defined as

$$\begin{aligned} ARI=\frac{\sum _{ij}\genfrac(){0.0pt}1{n_{ij}}{2}- \left[ \sum _i\genfrac(){0.0pt}1{a_i}{2}\sum _j\genfrac(){0.0pt}1{b_j}{2}\right] \genfrac(){0.0pt}1{n}{2}}{\frac{1}{2}\left[ \sum _i\genfrac(){0.0pt}1{a_i}{2}\sum _j\genfrac(){0.0pt}1{b_j}{2}\right] - \left[ \sum _i\genfrac(){0.0pt}1{a_i}{2} \sum _j\genfrac(){0.0pt}1{b_j}{2}\right] \genfrac(){0.0pt}1{n}{2}} \end{aligned}$$

where $n_{ij}$ denotes the number of shared cells between cluster *i* in ground truth and cluster *j* in clustering results, *n* denotes the number of all the cells, $a_i=\sum _jn_{ij}$ and $b_j=\sum _in_{ij}$. We visualized the consistency by projecting the original data and imputed data into two dimensions, with different colors of points representing different labels of ground truth.

We used silhouette coefficient to measure coherency, based on the ground truth or clusters generated using SC3. The silhouette score of a sample *i* is defined as

$$\begin{aligned} s(i)=\frac{b(i)-a(i)}{max(a(i),b(i))} \end{aligned}$$

where *a*(*i*) denotes the mean intra-cluster distance of sample *i* and *b*(*i*) is the mean nearest-cluster distance of sample *i*. The silhouette score of a clustering is the mean silhouette score of all of the samples.

For real datasets, clusters annotated in the corresponding study were used as the ground truth. For simulated datasets, the original clusters in the baseline dataset were used as the ground truth.

### Marker gene analysis

To determine whether imputation can improve marker gene analysis, we used marker genes to discriminate cell clusters for data before and after imputation, and evaluated whether the clusters are well separated. Marker genes of each cell type and subset were extracted from the published literature.

First, the mean expression value of marker genes was used to measure how much a cell belongs to a cell type. For a particular cell type, if the mean value of the corresponding marker gene expression in a cell exceeded a predefined threshold, the cell would be labeled with that cell type. A cell could have no label, one label or multiple labels. The predefined threshold was optimized to maximize the proportion of one-label cells, while ensuring that more than 90% of cells had labels. The threshold varied among different datasets and imputation methods, due to their heterogeneity.

Based on the cell labels, a new dataset was then constructed from the original dataset. The constructed dataset consisted of the two-dimensional projection of cells with one or more labels. Multi-labeled cells were duplicated multiple times. The silhouette score was calculated for the constructed datasets, to evaluate whether they could be well separated by the labels.

### Settings of imputation methods

The type of input data and the parameter settings of all of the imputation methods are shown in Table 2.

**Table 5:** R2 score on raw count datasets

Dataset	raw$^{*}$	SIMLR	ZINB-WaVE	scImpute	DrImpute	SAVER	MAGIC	NE	scVI	DCA	scScope	SAUCIE
ILC	$-$0.01	0.31	$-$0.01	0.20	0.52	$-$0.01	0.42	0.58	$-$1.35	0.71	$-$0.01	0.45
HCC	$-$0.09	0.22	$-$0.05	$-$9.07	0.54	$-$0.09	0.35	0.61	$-$3.79	0.76	$-$0.09	0.46
CRC	$-$0.07	0.13	$-$0.04	$-$3.74	0.59	$-$0.07	0.31	0.65	0.73	0.77	0.40	0.38
NSCLC	$-$0.07	0.11	$-$0.04	$-$5.51	0.56	$-$0.07	0.31	0.59	$-$0.02	0.77	0.37	0.38
PBMC	$-$0.22	0.18	$-$0.24	$-$2.93	0.70	0.00	0.64	0.73	0.82	0.80	0.55	0.63
BCC	$-$0.05	0.14	$-$1.92	$-$	$-$	$-$0.03	0.09	0.35	0.20	0.22	0.11	0.13
ITC	$-$0.11	0.05	$-$0.17	0.16	0.81	0.04	0.95	0.82	0.67	0.85	$-$0.08	0.28
DC.human	$-$0.07	0.23	$-$0.07	$-$0.05	0.90	0.02	0.96	0.87	0.81	0.94	$-$0.04	0.55
DC.mouse	$-$0.06	0.23	$-$0.06	$-$1.23	0.75	0.00	0.94	0.78	0.80	0.90	0.97	0.61
Melanoma.1	$-$0.06	0.23	$-$0.09	$-$12.01	0.48	$-$0.01	0.48	0.67	0.65	0.65	0.01	0.44
Sim1	$-$0.14	0.24	$-$0.71	0.60	0.84	$-$0.13	0.82	0.77	$-$773	0.96	0.96	0.74
Sim2	$-$0.19	0.22	$-$0.57	0.59	0.84	$-$0.18	0.82	0.80	$-$99.2	0.96	0.80	0.79
Sim3	$-$0.25	0.16	$-$0.41	0.52	0.81	$-$0.24	0.80	0.75	$-$1.99	0.93	0.76	0.75
Sim4	$-$0.48	$-$0.02	$-$0.46	$-$0.10	0.74	$-$0.48	0.71	0.46	0.47	0.91	0.91	0.53

$^{*}$ ’raw’ indicates data before imputation

$$^{**}$$ ’-’ means a method failed to finish imputation

Specially, the NE algorithm, a network enhancement method [14], was adapted to impute scRNA-seq data for the first time in this study. Firstly, we normalized the input data by

$$\begin{aligned} \log _2 (\frac{10^5*C_{ij}}{\sum _i C_{ij}}+1) \end{aligned}$$

where $C_{ij}$ is the expression value of gene *i* in cell *j*. Next, we constructed a cell-to-cell similarity matrix by calculating the Pearson correlation between cells. Network enhancement was then applied to denoise the similarity matrix and enhance the cell-to-cell correlations. We normalized the denoised similarity matrix to a weighting matrix by dividing all of the similarity values by the maximum value of each cell, and set the self-weight of each cell to 1.5 times the maximum weight to its neighbors. To share information from similar cells, recovered data were obtained by multiplying the weighting matrix by the original data matrix, as in MAGIC [13].

For SIMLR [15], we first used the method to directly learn a cell-to-cell similarity matrix from the input data. The similarity matrix was then normalized to a weighting matrix, in which the sum of each row equaled one. We then multiplied the weighting matrix by the input data matrix to obtain the imputed data.

When using SAUCIE [10], the input data were first reduced to 100 dimensions by PCA before imputation. The output data were then inversely converted to the original dimensions to give the imputation results.

## Supplementary information

**Additional file 1: Fig. S1**: Visualization of different methods on ILC. On dataset ILC, data before imputation ('raw') and after imputed by different methods were visualized by t-SNE, with different colors representing different cell types. For each subgraph, values in the lower left and lower right corners represent the silhouette scores based on the ground truth and the ARI scores, respectively.

**Additional file 2: Fig. S2** Visualization of different methods on PBMC. On dataset PBMC, data before imputation ('raw') and after imputed by different methods were visualized by t-SNE, with different colors representing different cell types. For each subgraph, values in the lower left and lower right corners represent the silhouette scores based on the ground truth and the ARI scores, respectively.

**Additional file 3: Fig. S3**Visualization of different methods on Melanoma.2. On dataset Melanoma.2, data before imputation ('raw') and after imputed by different methods were visualized by t-SNE, with different colors representing different cell types. For each subgraph, values in the lower left and lower right corners represent the silhouette scores based on the ground truth and the ARI scores, respectively.

**Additional file 4: Fig. S4** Visualization of different methods on HCC. On dataset HCC, data before imputation ('raw') and after imputed by different methods were visualized by t-SNE, with different colors representing different cell types. For each subgraph, values in the lower left and lower right corners represent the silhouette scores based on the ground truth and the ARI scores, respectively.

**Additional file 5: Fig. S5** Visualization of different methods on NSCLC. On dataset NSCLC, data before imputation ('raw') and after imputed by different methods were visualized by t-SNE, with different colors representing different cell types. For each subgraph, values in the lower left and lower right corners represent the silhouette scores based on the ground truth and the ARI scores, respectively.

**Additional file 6: Fig. S6** Visualization of different methods on DC_mouse. On dataset DC_mouse, data before imputation ('raw') and after imputed by different methods were visualized by t-SNE, with different colors representing different cell types. For each subgraph, values in the lower left and lower right corners represent the silhouette scores based on the ground truth and the ARI scores, respectively.

**Additional file 7: Fig. S7** Visualization of raw count, SAVER, NE and AutoImpute. Data before imputation ('raw') and after imputed by SAVER, NE, and AutoImpute were visualized by t-SNE, with different colors representing different cell types. For these data, log transformation was not performed before visualization, as AutoImpute imputed data with many negative values.

**Additional file 8: Fig. S8** The performance of five imputation methods on benchmarking datasets.Five selected imputation methods (scImpute, DrImpute, SAVER, NE, and DCA) were tested on datasets from three different protocols (Quartz-Seq2, Chromium, and Smart-Seq2). (a) For the numerical recovery task, two indices, the median error and mean error, are shown. (b) For clustering analysis, three indices, ARI, silhouette based on ground truth 'sil(g)', and silhouette based on SC3 clusters 'sil(s)' are shown. The five selected imputation methods did not show different tendencies with respect to these five indices across these three protocols. Human samples including PBMCs and HEK293T cells, were used for the analyses.

**Additional file 9: Fig. S9** Cluster visualization of five imputation methods on benchmarking datasets.Clusters on three different protocols (Quartz-Seq2, Chromium, and Smart-Seq2) were visualized by t-SNE. Colored cell labels were directly derived from the original study.

**Additional file 10: Fig. S10** Marker gene expression on HCC, CRC, NSCLC and DC_human. Expression values of marker genes of different datasets before and after imputation are shown: CD3D and CD3E for HCC, CRC and NSCLC; ITGAX and CD1C for DC_human. Expression values of marker genes in different datasets before and after imputation are shown. We selected the following marker genes for analysis: CD3D and CD3E for T cells; CD79A and CD79B for B cells; NCAM1 for NK cells; and ITGAX and CD1C for DCs.The datasets HCC, CRC, and NSCLC represent T cells, which should highly express CD3D and CD3E, but not CD79A, CD79B, or NCAM1. The dataset DC_human represents DCs, which should highly express ITGAX and CD1C, but not CD3E, CD79A, or CD79B.

**Additional file 11: Fig. S11** Marker gene expression of different cell types from the Quartz-Seq2 protocol. Expression values of marker genes of different cell types are shown: CD3D and CD4 for CD4$^+$ T cells; CD3D, CD8A and CD8B for CD8$^+$ T cells; CD19 and CD79A for B cells; NCAM1 for NK cells;CD14 for CD14$^+$ monocytes; FCGR3A for FCGR3A$^+$ monocytes; SLIT2 for HEK293T cells.

**Additional file 12: Fig. S12** Marker gene expression of different cell types from the Chromium protocol.Expression values of marker genes of different cell types are shown:CD3D and CD4 for CD4$^+$ T cells; CD3D, CD8A and CD8B for CD8$^+$ T cells; CD19 and CD79A for B cells; NCAM1 for NK cells; CD14 for CD14$^+$ monocytes; FCGR3A for FCGR3A$^+$ monocytes; SLIT2 for HEK293T cells.

**Additional file 13: Fig. S13** Marker gene expression of different cell types from the Smart-Seq2 protocol. Expression values of marker genes of different cell types are shown: CD3D and CD4 for CD4$^+$ T cells; CD3D, CD8A and CD8B for CD8$^+$ T cells; CD19 and CD79A for B cells; NCAM1 for NK cells;CD14 for CD14$^+$ monocytes; FCGR3A for FCGR3A$^+$ monocytes; SLIT2 for HEK293T cells.

**Additional file 14: Fig. S14** Run time of different imputation methods. The run times (in seconds) of different methods are shown for all datasets. Green and yellow grids correspond to faster and slower speeds, respectively. The methods were ranked by the 'overall' score, which is the average score of all of the datasets.

## Data Availability

The detailed list of datasets used in the current study is described in the “Methods” section. All the codes are available at https://github.com/Joye9285/Imputation-benchmark.

## Abbreviations

scRNA-seq: Single-cell RNA sequencing
ILC: Innate lymphoid cell
HCC: Hepatocellular carcinoma
CRC: Colorectal cancer
NSCLC: Non-small cell lung cancer
PBMC: Peripheral blood mononuclear cell
BCC: Basal cell carcinoma
ITC: Innate T cell
DC: Dendritic cell
BRCA: Breast cancer
SIMLR: Single-cell interpretation via multi-kernel learning
ZINBWaVE: Zero-inflated negative binomial-based wanted variation extraction
SAVER: Single-cell analysis via expression recovery
MAGIC: Markov affinity-based graph imputation of cells
NE: Network enhancement
scVI: Single-cell variational inference
DCA: Deep count autoencoder network
SAUCIE: Sparse autoencoder for unsupervised clustering, imputation and embedding
TPM: Transcripts per kilobase million
SC3: Single-cell consensus clustering
ARI: Adjusted rand index
NK: Natural killer
UMI: Unique molecular identifier
IQR: Interquartile range
t-SNE: T-distributed stochastic neighbor embedding
PCA: Principal component analysis
CD3D: Cluster of differentiation antigen 3d molecule
CD3E: CD3e molecule
CD19: CD19 molecule
CD79A: CD79a molecule
CD56: CD56 molecule, also known as neural cell adhesion molecule 1/NCAM1
GZMH: Granzyme H
CD14: CD14 molecule
ITGAM: Integrin subunit alpha M
CD16: CD16 molecule, also known as Fc fragment of IgG receptor IIIa/FCGR3A
ITGAX: Integrin subunit alpha X
CD1C: CD1c molecule
CD79B: CD79b molecule
CD8A: CD8a molecule
CD8B: CD8b molecule
CD4: CD4 molecule
SLIT2: Slit guidance ligand 2
