# Supplementary figures and images for: Evaluating imputation methods for single-cell RNA-seq data
Source: BMC Bioinformatics. 2023 Jul 28;24:302. doi: 10.1186/s12859-023-05417-7 (PMC10386301; doi:10.1186/s12859-023-05417-7)

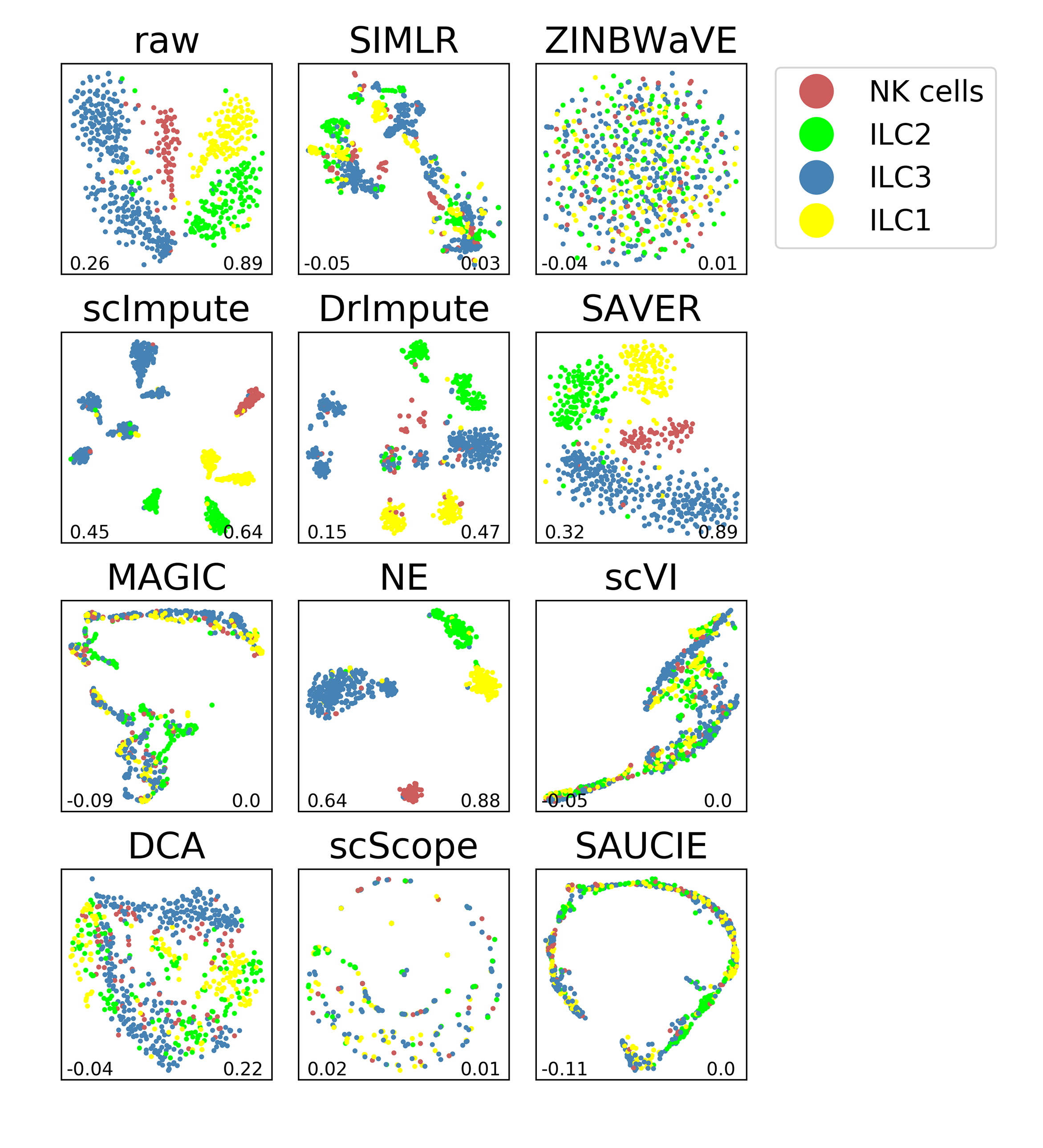

Supplement: Supplementary file 1 — Additional file 1: Fig. S1: Visualization of different methods on ILC. On dataset ILC, data before imputation ('raw') and after imputed by different methods were visualized by t-SNE, with different colors representing different cell types. For each subgraph, values in the lower left and lower right corners represent the silhouette scores based on the ground truth and the ARI scores, respectively. [file 12859_2023_5417_MOESM1_ESM.png]

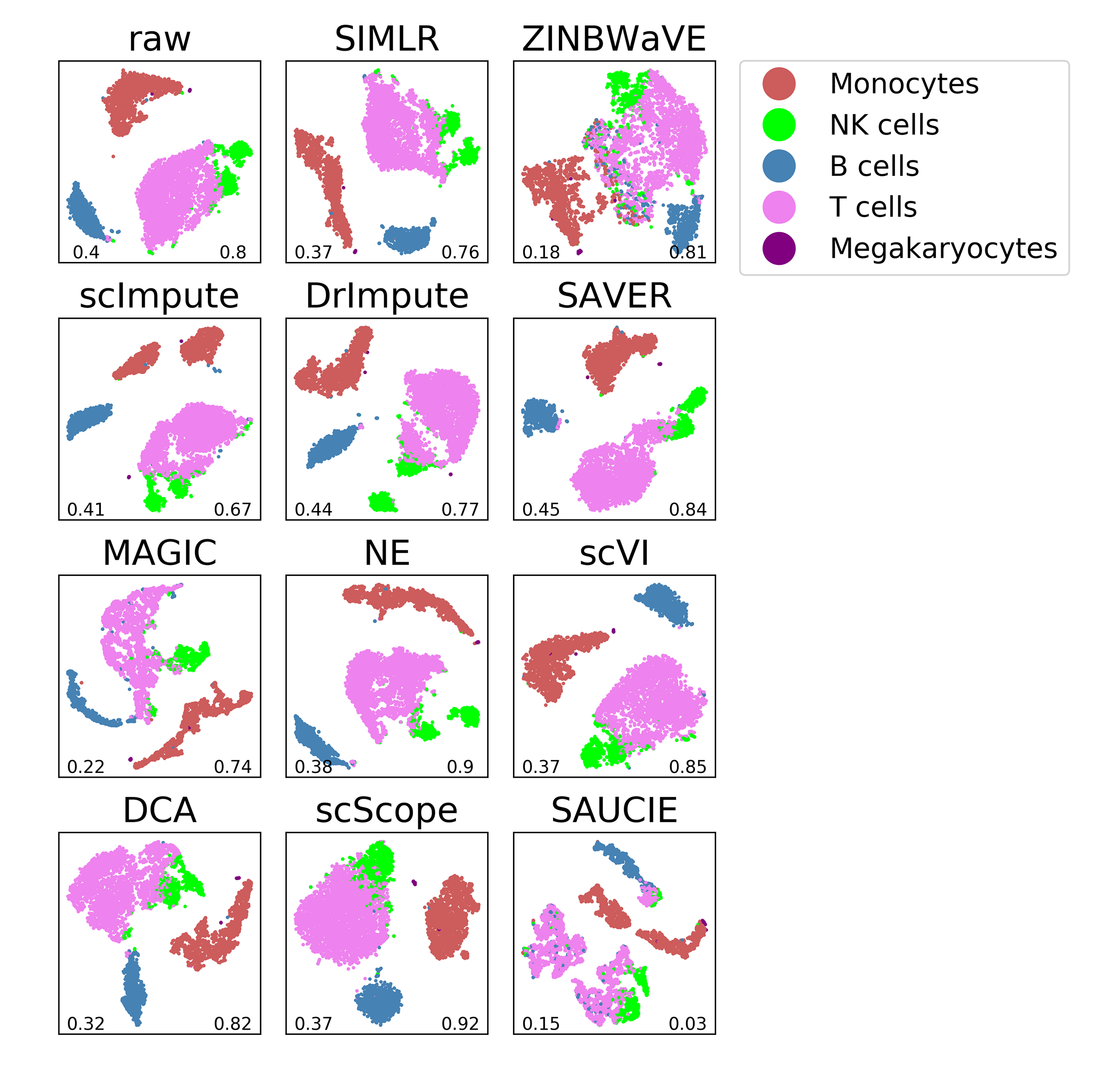

Supplement: Supplementary file 2 — Additional file 2: Fig. S2 Visualization of different methods on PBMC. On dataset PBMC, data before imputation ('raw') and after imputed by different methods were visualized by t-SNE, with different colors representing different cell types. For each subgraph, values in the lower left and lower right corners represent the silhouette scores based on the ground truth and the ARI scores, respectively. [file 12859_2023_5417_MOESM2_ESM.png]

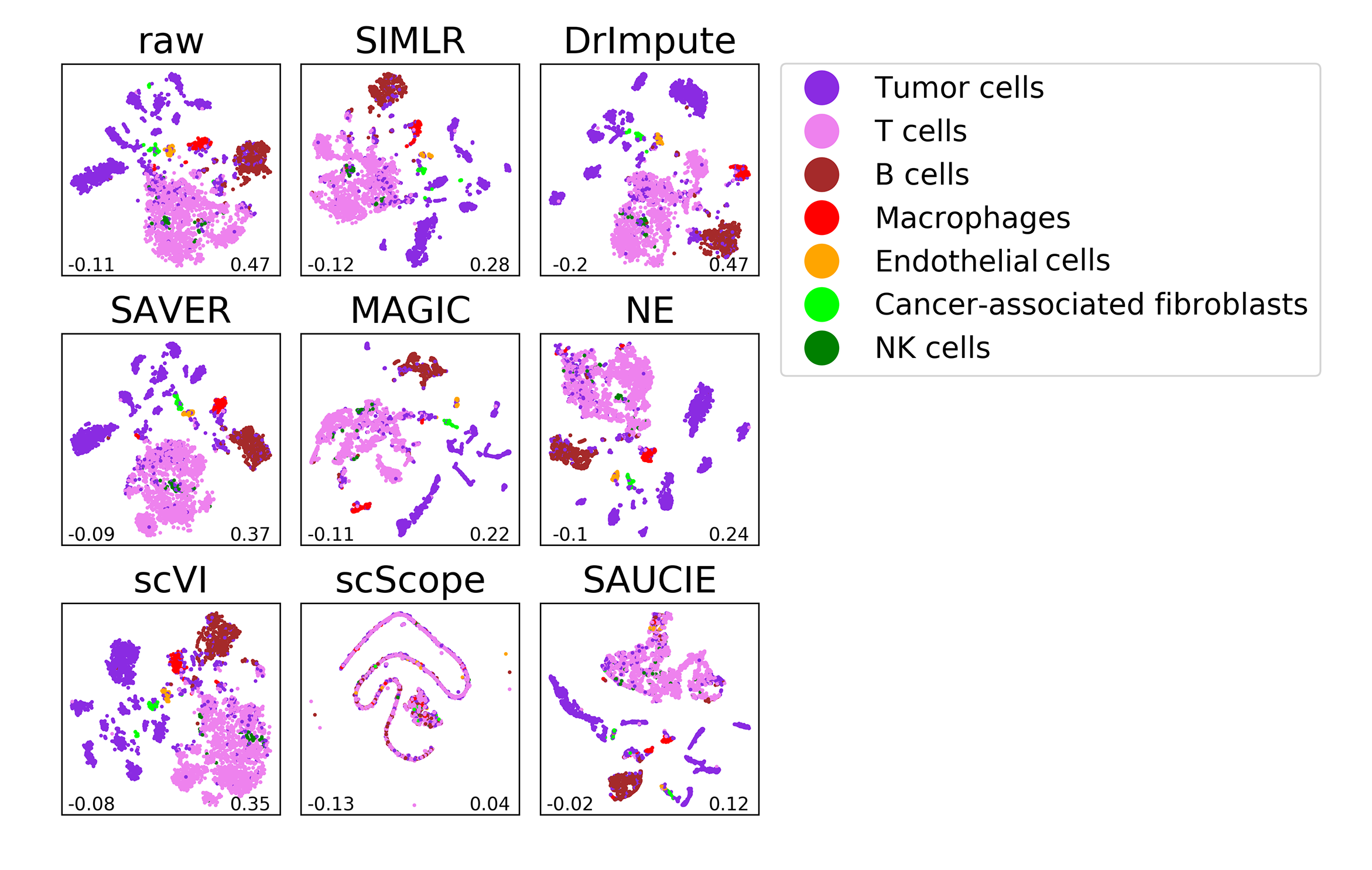

Supplement: Supplementary file 3 — Additional file 3: Fig. S3Visualization of different methods on Melanoma.2. On dataset Melanoma.2, data before imputation ('raw') and after imputed by different methods were visualized by t-SNE, with different colors representing different cell types. For each subgraph, values in the lower left and lower right corners represent the silhouette scores based on the ground truth and the ARI scores, respectively. [file 12859_2023_5417_MOESM3_ESM.png]

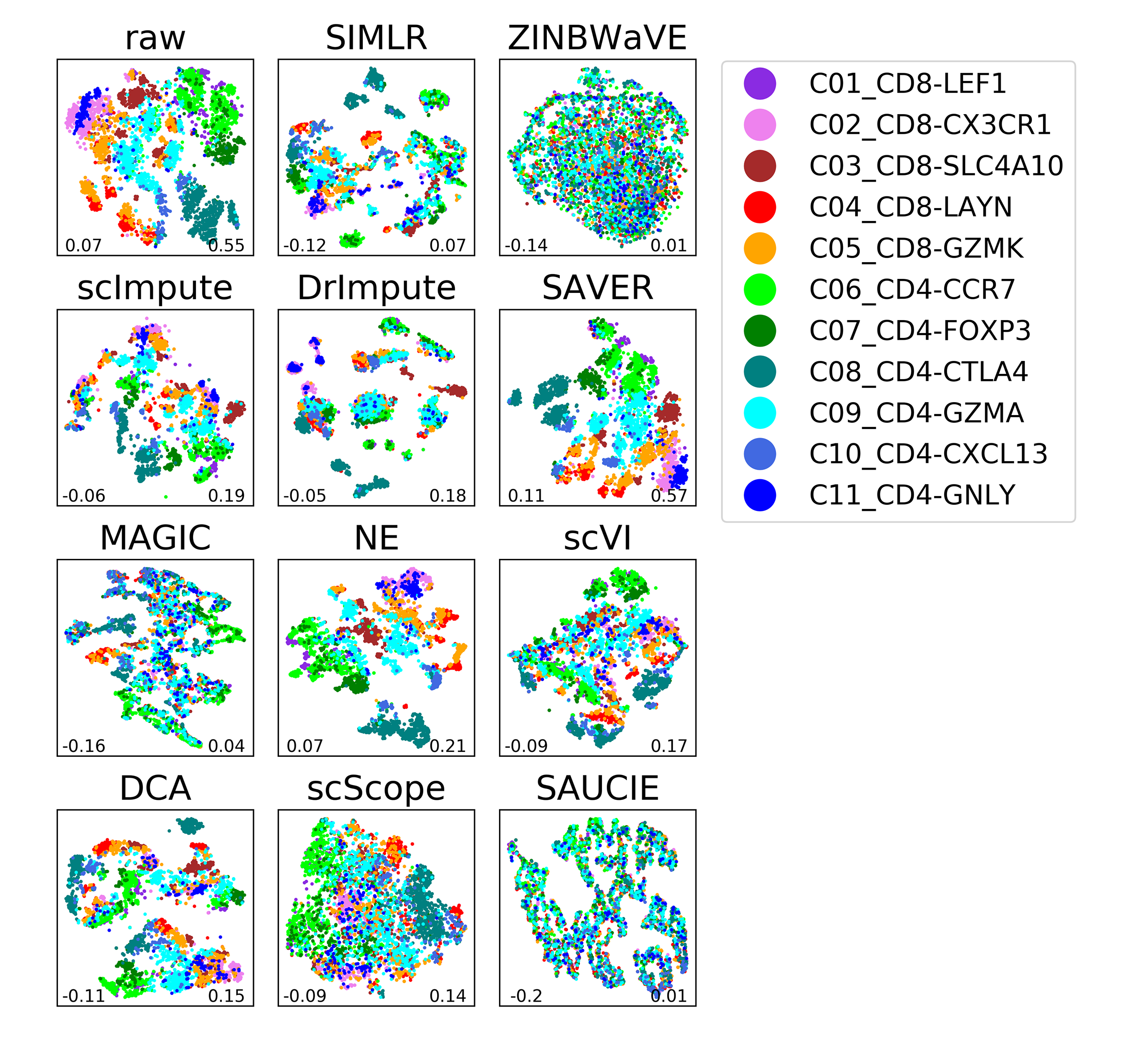

Supplement: Supplementary file 4 — Additional file 4: Fig. S4 Visualization of different methods on HCC. On dataset HCC, data before imputation ('raw') and after imputed by different methods were visualized by t-SNE, with different colors representing different cell types. For each subgraph, values in the lower left and lower right corners represent the silhouette scores based on the ground truth and the ARI scores, respectively. [file 12859_2023_5417_MOESM4_ESM.png]

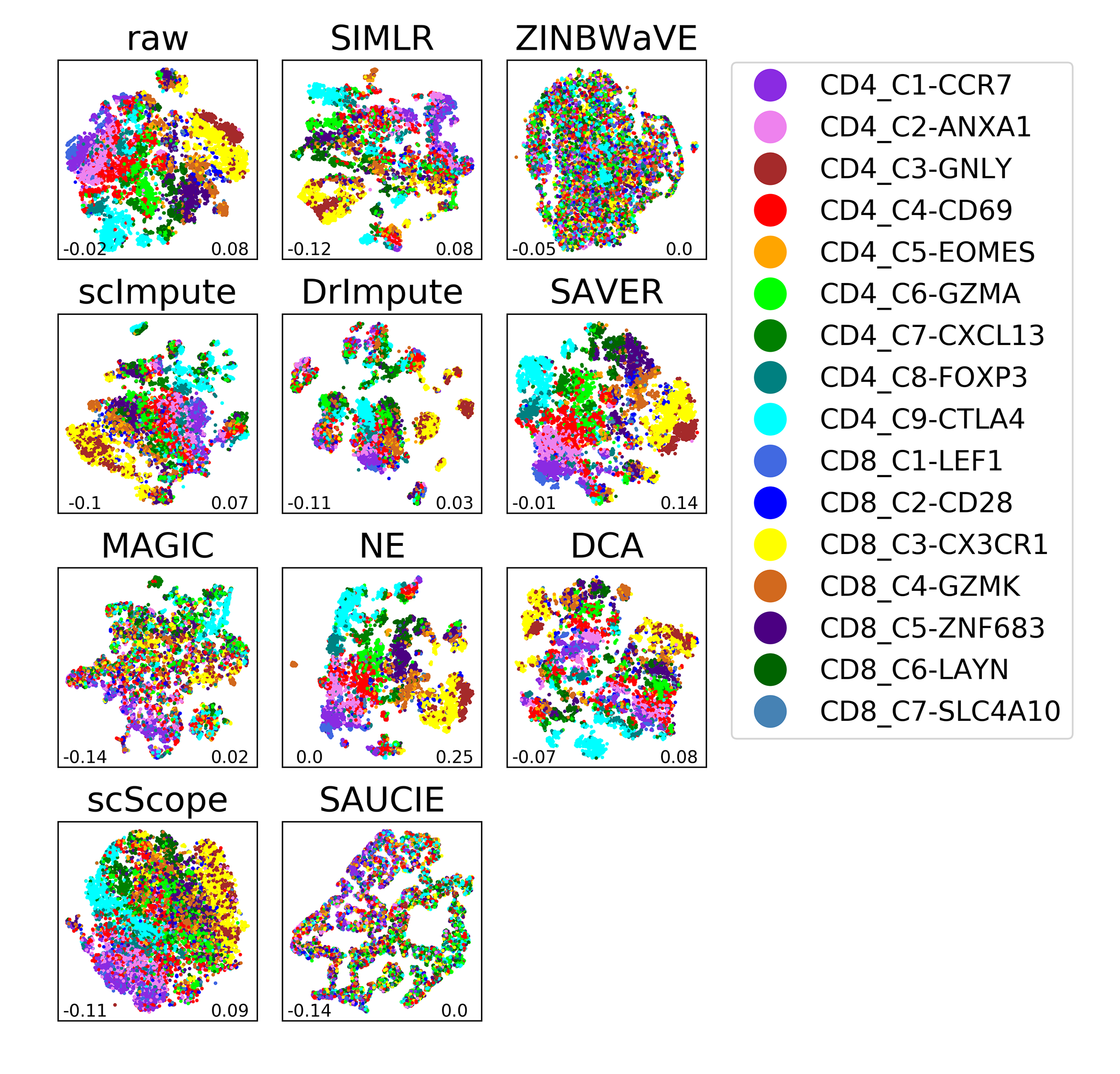

Supplement: Supplementary file 5 — Additional file 5: Fig. S5 Visualization of different methods on NSCLC. On dataset NSCLC, data before imputation ('raw') and after imputed by different methods were visualized by t-SNE, with different colors representing different cell types. For each subgraph, values in the lower left and lower right corners represent the silhouette scores based on the ground truth and the ARI scores, respectively. [file 12859_2023_5417_MOESM5_ESM.png]

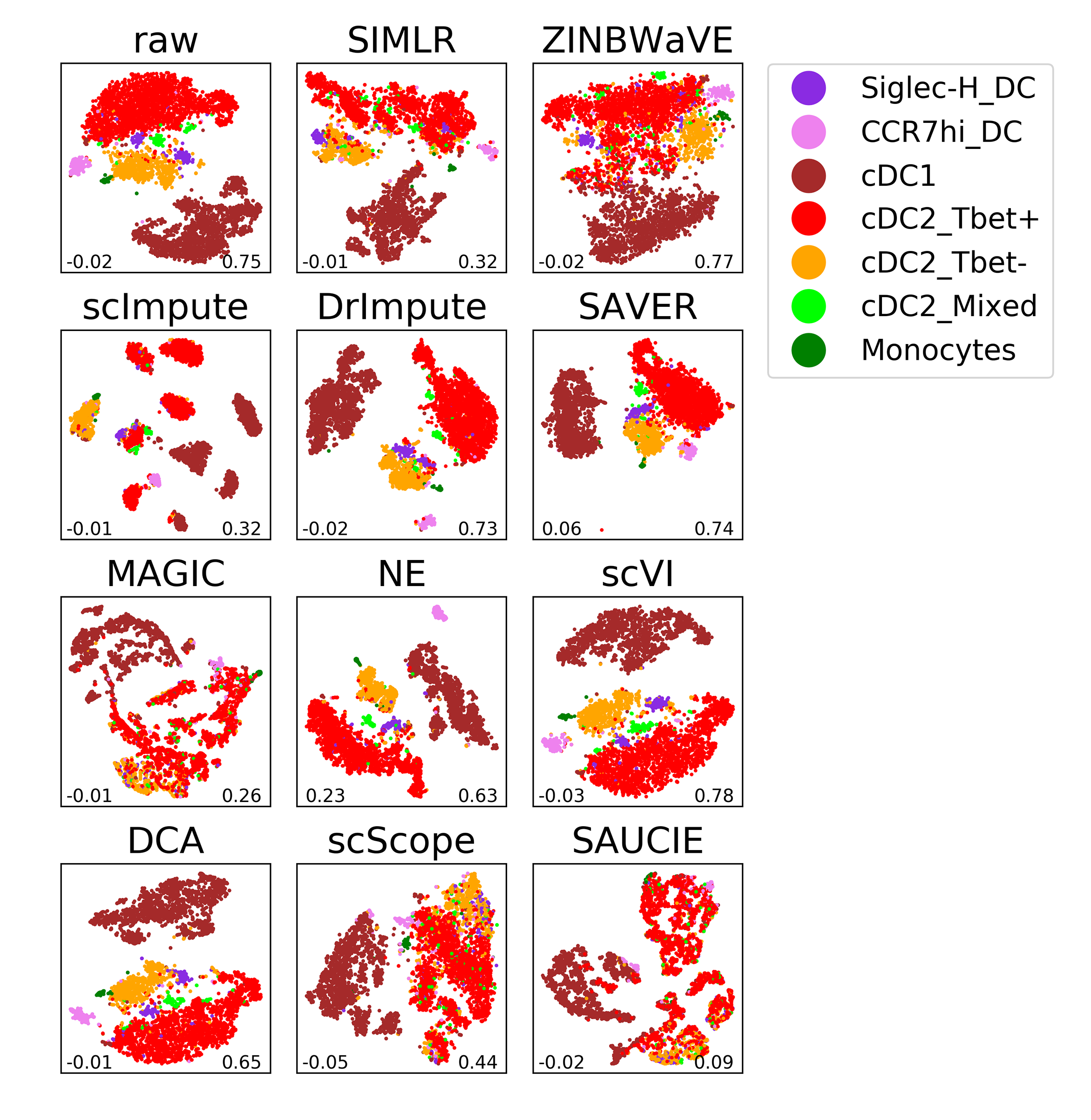

Supplement: Supplementary file 6 — Additional file 6: Fig. S6 Visualization of different methods on DC_mouse. On dataset DC_mouse, data before imputation ('raw') and after imputed by different methods were visualized by t-SNE, with different colors representing different cell types. For each subgraph, values in the lower left and lower right corners represent the silhouette scores based on the ground truth and the ARI scores, respectively. [file 12859_2023_5417_MOESM6_ESM.png]

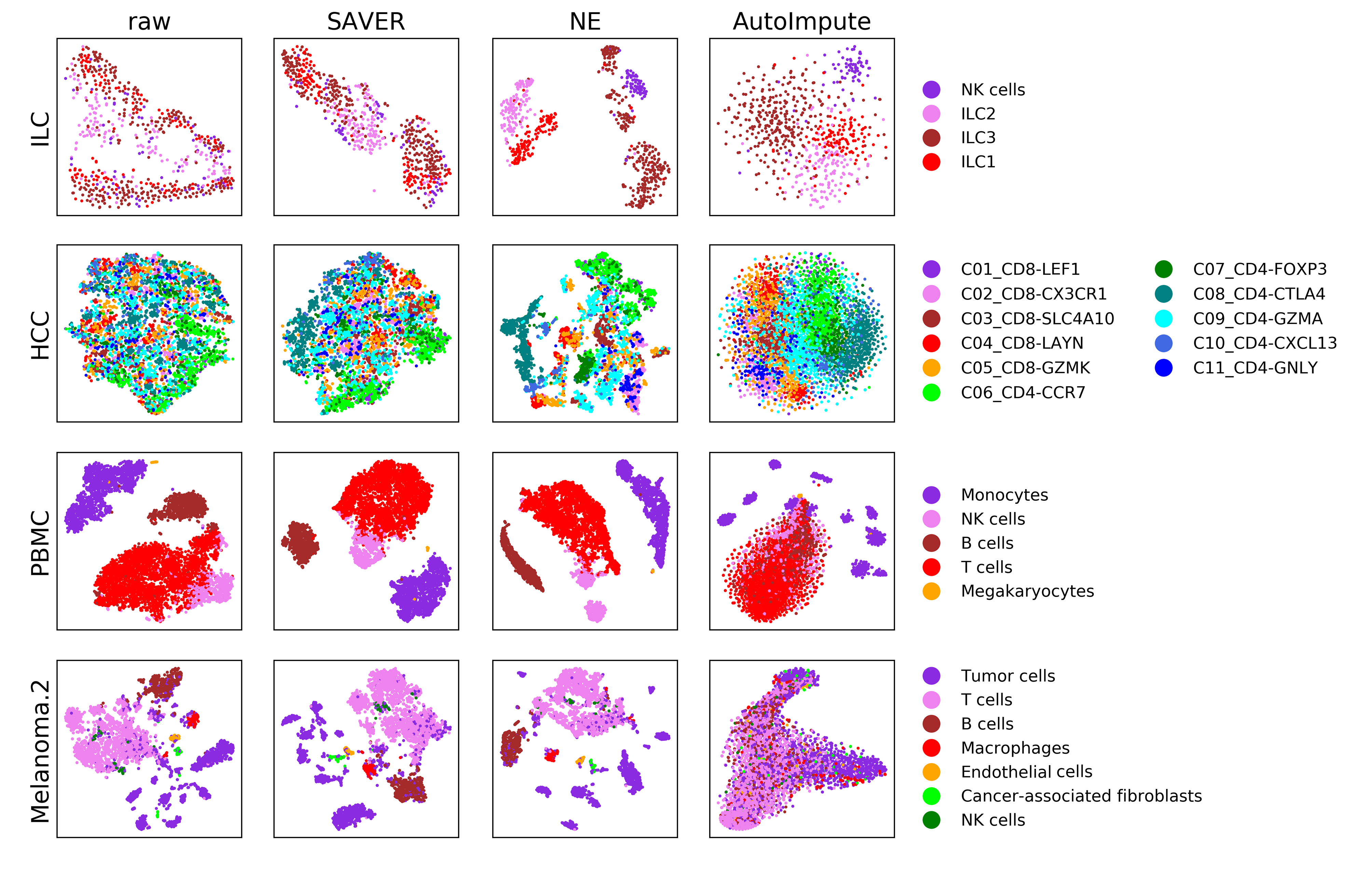

Supplement: Supplementary file 7 — Additional file 7: Fig. S7 Visualization of raw count, SAVER, NE and AutoImpute. Data before imputation ('raw') and after imputed by SAVER, NE, and AutoImpute were visualized by t-SNE, with different colors representing different cell types. For these data, log transformation was not performed before visualization, as AutoImpute imputed data with many negative values. [file 12859_2023_5417_MOESM7_ESM.png]

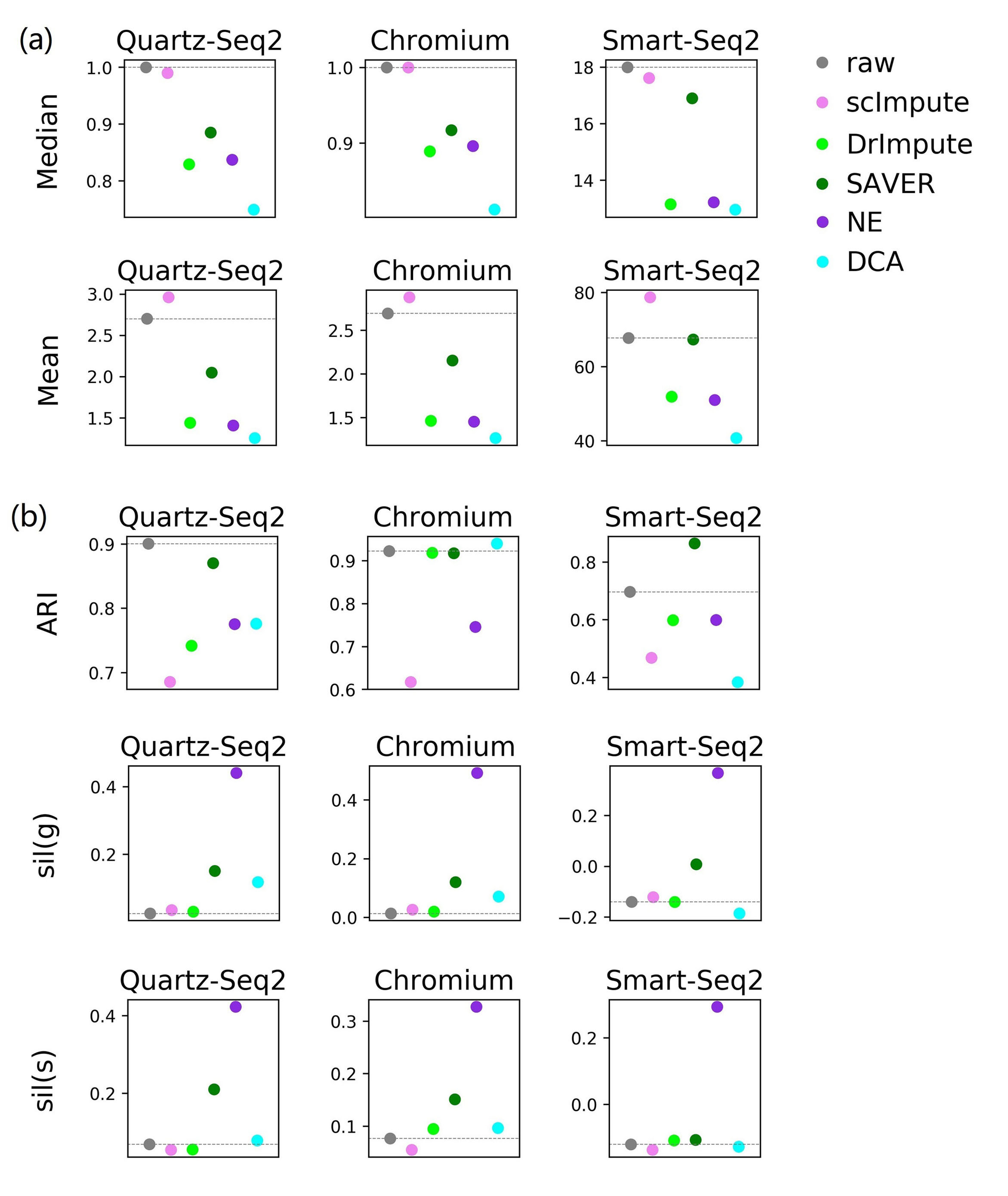

Supplement: Supplementary file 8 — Additional file 8: Fig. S8 The performance of five imputation methods on benchmarking datasets.Five selected imputation methods (scImpute, DrImpute, SAVER, NE, and DCA) were tested on datasets from three different protocols (Quartz-Seq2, Chromium, and Smart-Seq2). (a) For the numerical recovery task, two indices, the median error and mean error, are shown. (b) For clustering analysis, three indices, ARI, silhouette based on ground truth 'sil(g)', and silhouette based on SC3 clusters 'sil(s)' are shown. The five selected imputation methods did not show different tendencies with respect to these five indices across these three protocols. Human samples including PBMCs and HEK293T cells, were used for the analyses. [file 12859_2023_5417_MOESM8_ESM.jpg]

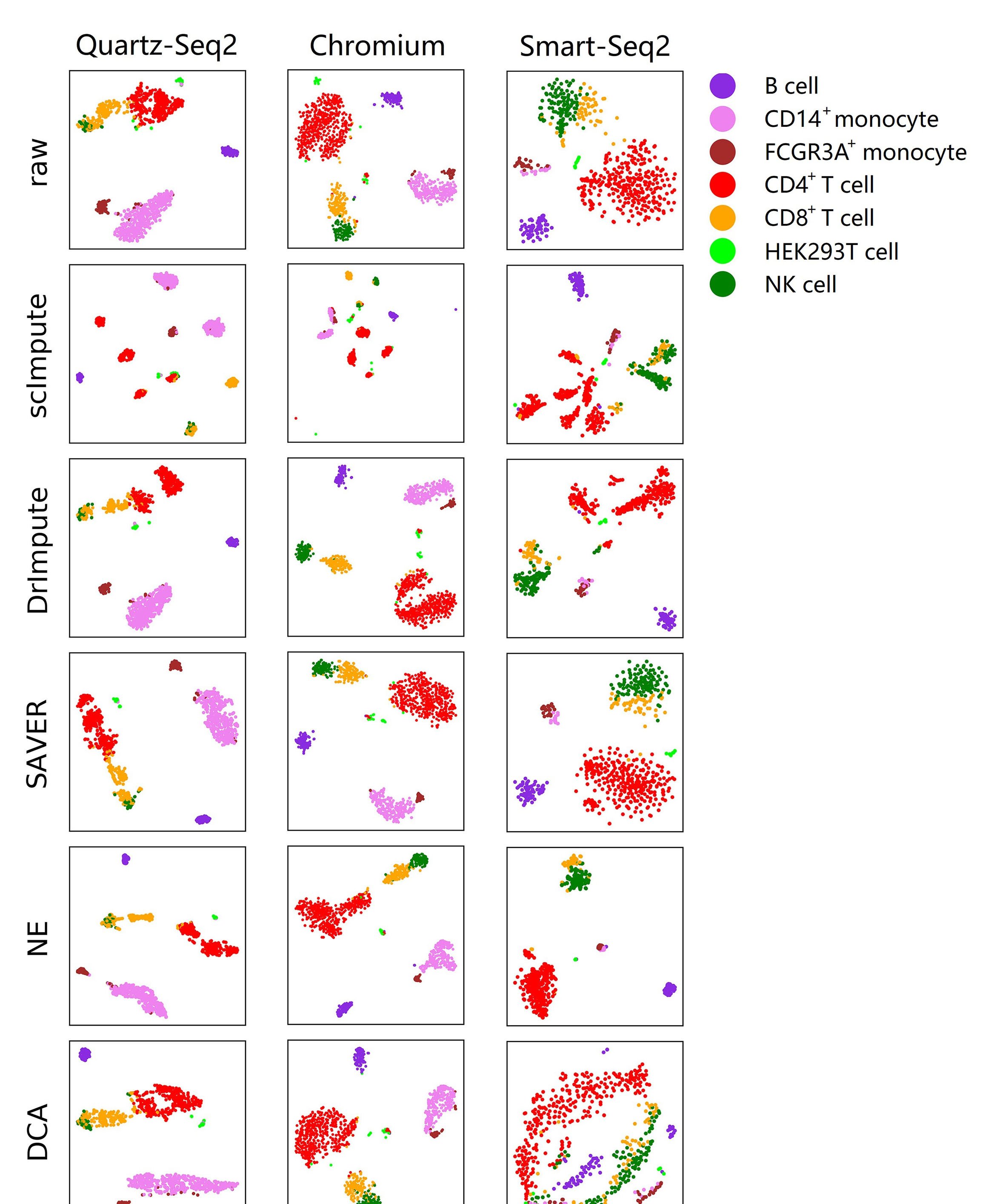

Supplement: Supplementary file 9 — Additional file 9: Fig. S9 Cluster visualization of five imputation methods on benchmarking datasets.Clusters on three different protocols (Quartz-Seq2, Chromium, and Smart-Seq2) were visualized by t-SNE. Colored cell labels were directly derived from the original study. [file 12859_2023_5417_MOESM9_ESM.jpg]

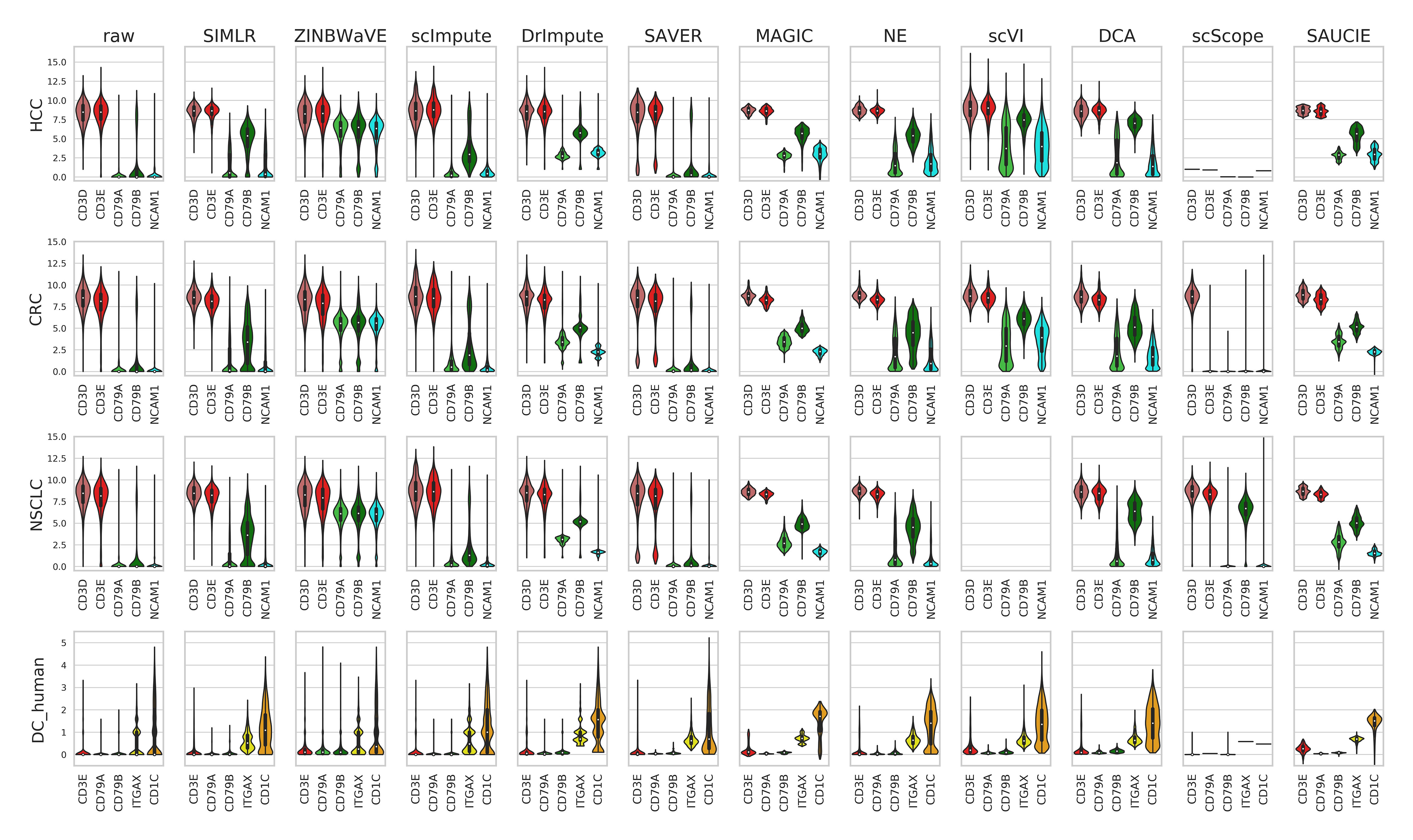

Supplement: Supplementary file 10 — Additional file 10: Fig. S10 Marker gene expression on HCC, CRC, NSCLC and DC_human. Expression values of marker genes of different datasets before and after imputation are shown: CD3D and CD3E for HCC, CRC and NSCLC; ITGAX and CD1C for DC_human. Expression values of marker genes in different datasets before and after imputation are shown. We selected the following marker genes for analysis: CD3D and CD3E for T cells; CD79A and CD79B for B cells; NCAM1 for NK cells; and ITGAX and CD1C for DCs.The datasets HCC, CRC, and NSCLC represent T cells, which should highly express CD3D and CD3E, but not CD79A, CD79B, or NCAM1. The dataset DC_human represents DCs, which should highly express ITGAX and CD1C, but not CD3E, CD79A, or CD79B. [file 12859_2023_5417_MOESM10_ESM.jpg]

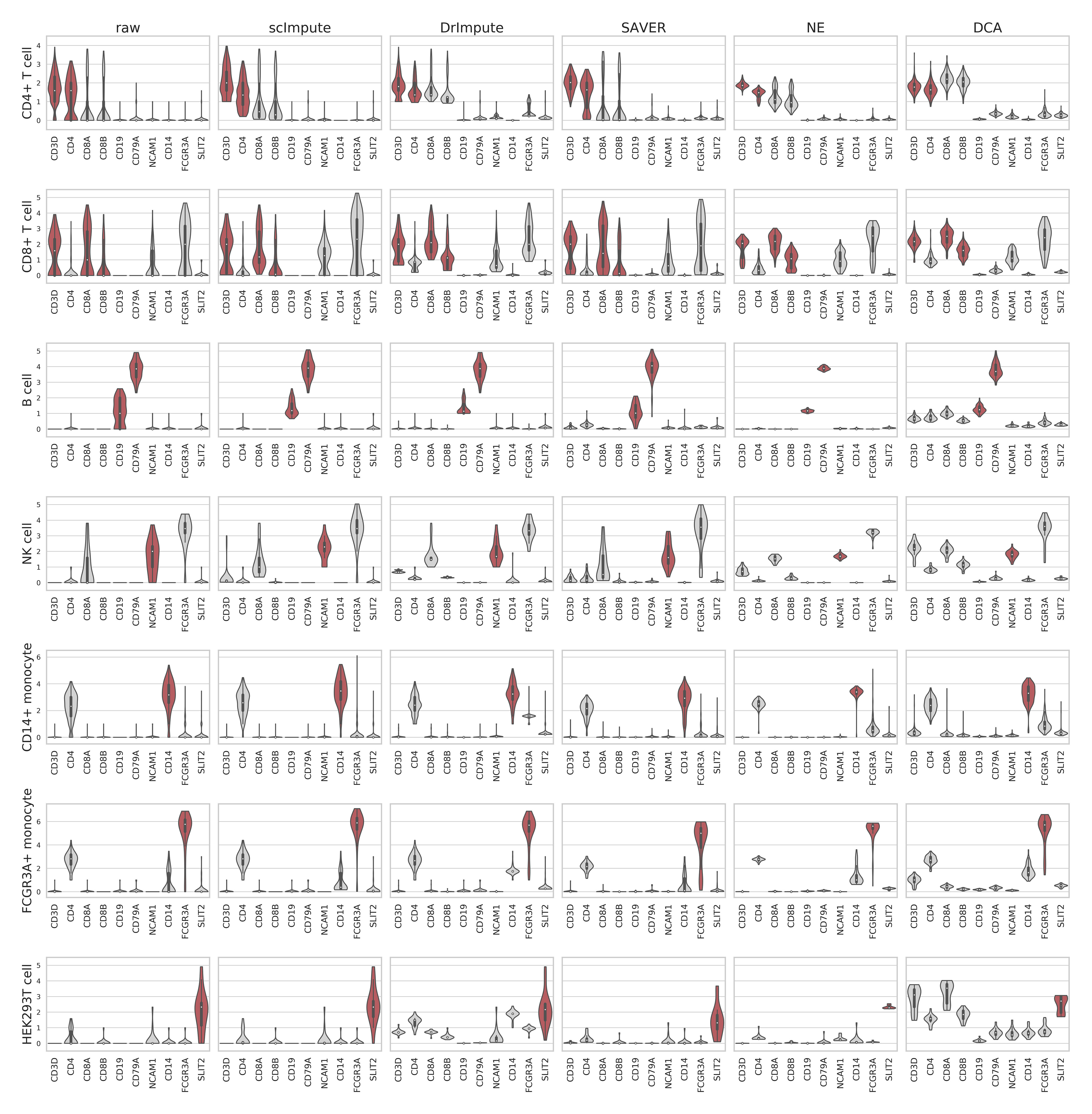

Supplement: Supplementary file 11 — Additional file 11: Fig. S11 Marker gene expression of different cell types from the Quartz-Seq2 protocol. Expression values of marker genes of different cell types are shown: CD3D and CD4 for CD4$^+$ T cells; CD3D, CD8A and CD8B for CD8$^+$ T cells; CD19 and CD79A for B cells; NCAM1 for NK cells;CD14 for CD14$^+$ monocytes; FCGR3A for FCGR3A$^+$ monocytes; SLIT2 for HEK293T cells. [file 12859_2023_5417_MOESM11_ESM.jpg]

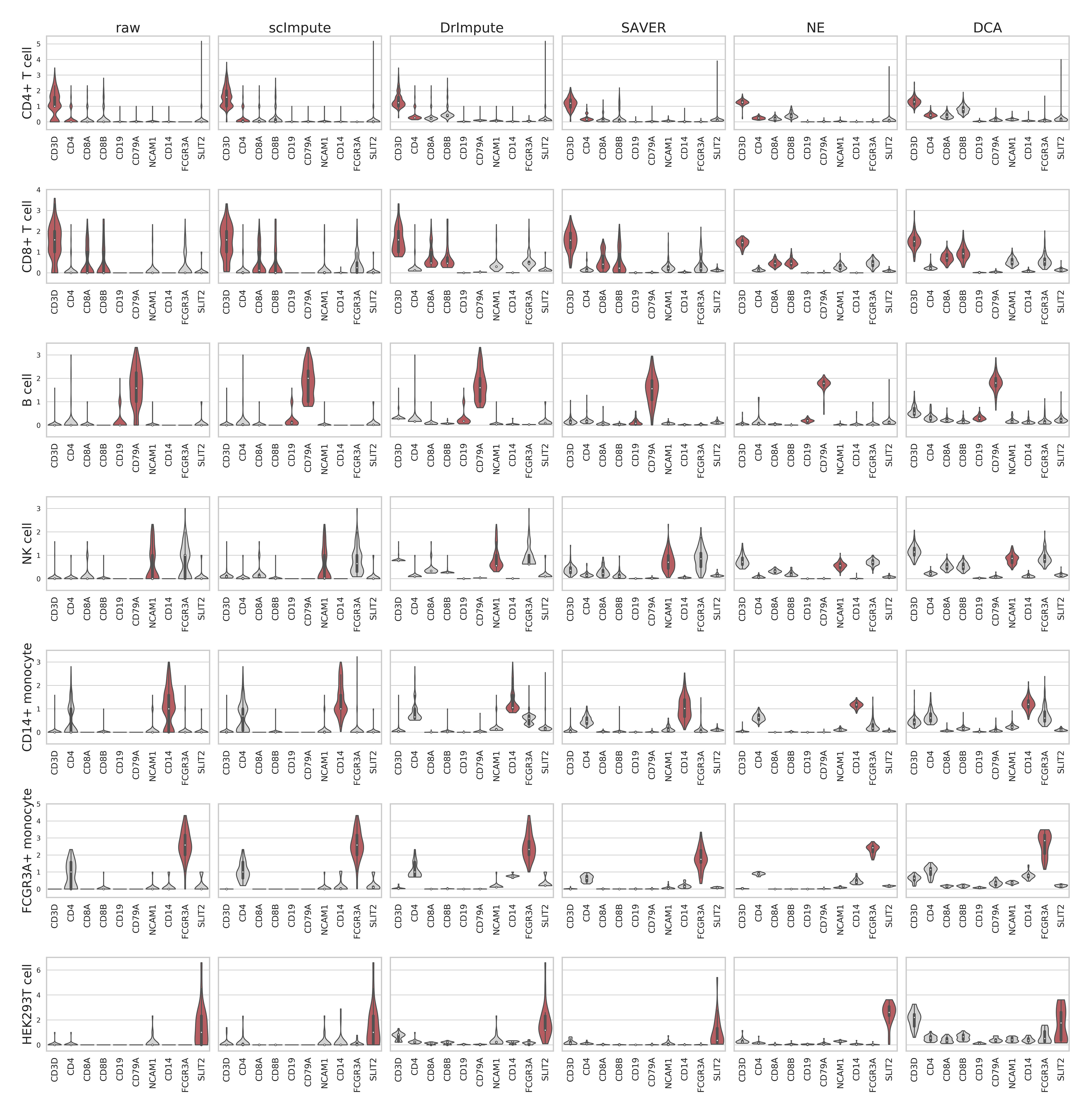

Supplement: Supplementary file 12 — Additional file 12: Fig. S12 Marker gene expression of different cell types from the Chromium protocol.Expression values of marker genes of different cell types are shown:CD3D and CD4 for CD4$^+$ T cells; CD3D, CD8A and CD8B for CD8$^+$ T cells; CD19 and CD79A for B cells; NCAM1 for NK cells; CD14 for CD14$^+$ monocytes; FCGR3A for FCGR3A$^+$ monocytes; SLIT2 for HEK293T cells. [file 12859_2023_5417_MOESM12_ESM.jpg]

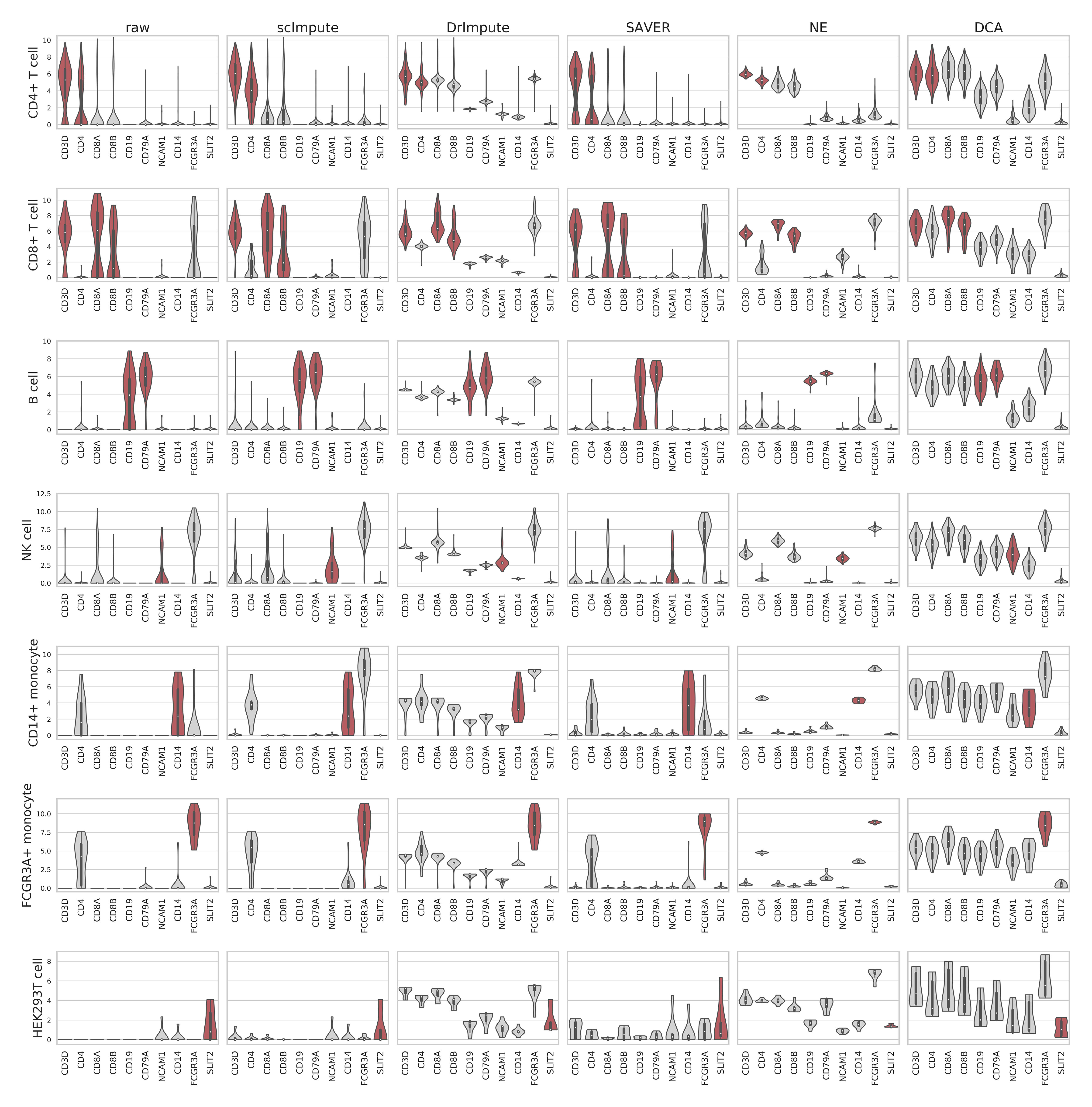

Supplement: Supplementary file 13 — Additional file 13: Fig. S13 Marker gene expression of different cell types from the Smart-Seq2 protocol. Expression values of marker genes of different cell types are shown: CD3D and CD4 for CD4$^+$ T cells; CD3D, CD8A and CD8B for CD8$^+$ T cells; CD19 and CD79A for B cells; NCAM1 for NK cells;CD14 for CD14$^+$ monocytes; FCGR3A for FCGR3A$^+$ monocytes; SLIT2 for HEK293T cells. [file 12859_2023_5417_MOESM13_ESM.jpg]

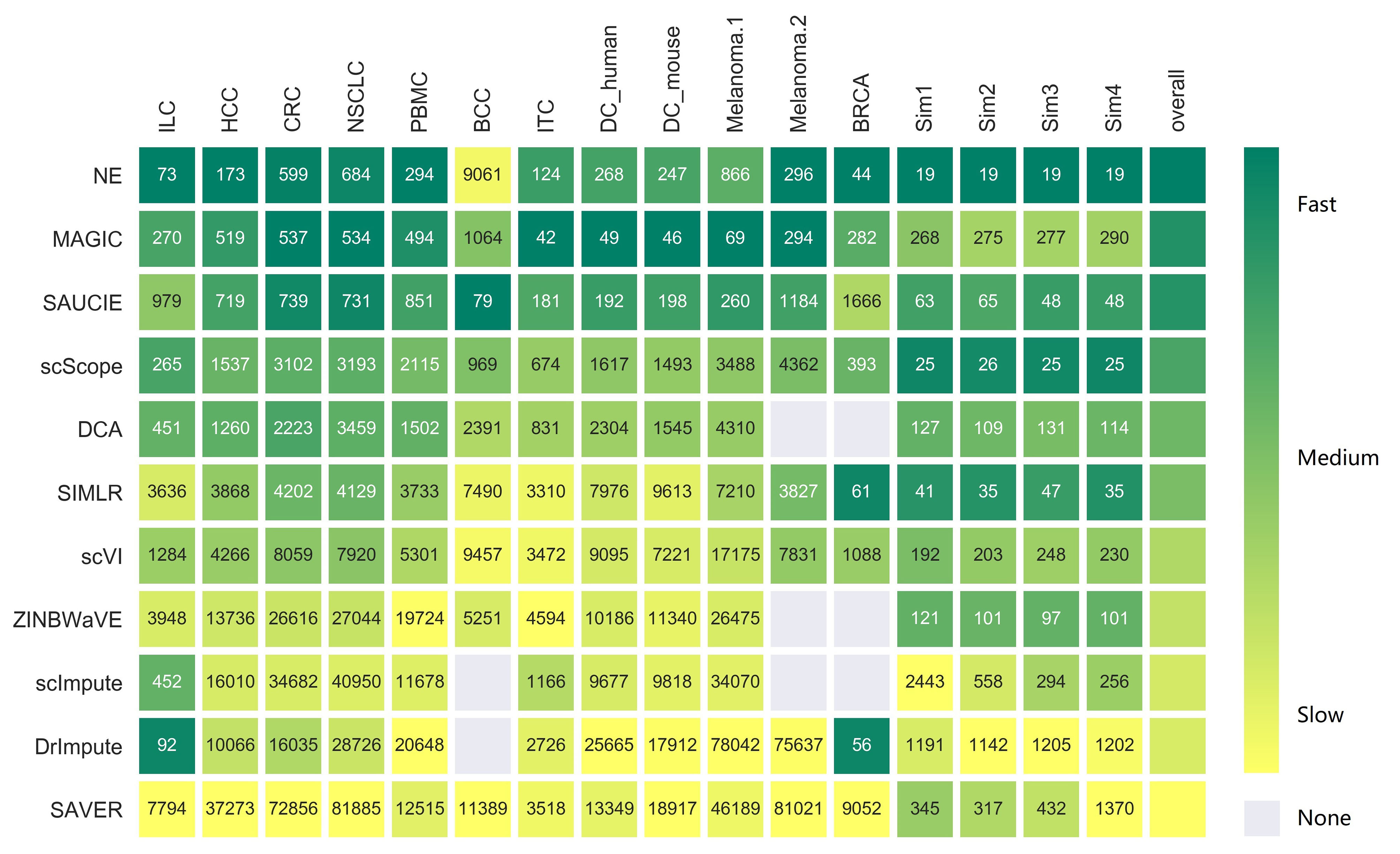

Supplement: Supplementary file 14 — Additional file 14: Fig. S14 Run time of different imputation methods. The run times (in seconds) of different methods are shown for all datasets. Green and yellow grids correspond to faster and slower speeds, respectively. The methods were ranked by the 'overall' score, which is the average score of all of the datasets. [file 12859_2023_5417_MOESM14_ESM.jpg]
